# Systematic review of patients’ and healthcare professionals’ views on patient‐initiated follow‐up in treated cancer patients

**DOI:** 10.1002/cam4.6243

**Published:** 2023-06-16

**Authors:** Janine Dretzke, Ava Lorenc, Ada Adriano, Clare Herd, Hisham Mehanna, Paul Nankivell, David J. Moore, the PETNECK2 Research Team, Andreas Karwath, Barry Main, Charlotte Firth, Claire Gaunt, Colin Greaves, Eila Watson, Georgios Gkoutos, Gozde Ozakinci, Jane Wolstenholme, Jo Brett, Joan Duda, Lauren Matheson, Louise‐Rae Cherrill, Melanie Calvert, Philip Kiely, Piers Gaunt, Saisakul Chernbumroong, Saloni Mittal, Steve Thomas, Stuart Winter, Wai Lup Wong

**Affiliations:** ^1^ Institute of Applied Health Research University of Birmingham Birmingham UK; ^2^ Bristol Medical School: Population Health Sciences, University of Bristol Bristol UK; ^3^ Institute of Head and Neck Studies and Education University of Birmingham Birmingham UK

**Keywords:** attitude, cancer, patient‐initiated follow‐up, qualitative research, survey, systematic review

## Abstract

**Background:**

Current follow‐up models in cancer are seen to be unsustainable and inflexible, and there is growing interest in alternative models, such as patient‐initiated follow‐up (PIFU). It is therefore important to understand whether PIFU is acceptable to patients and healthcare professionals (HCPs).

**Methods:**

Standard systematic review methodology aimed at limiting bias was used for study identification (to January 2022), selection and data extraction. Thematic synthesis was undertaken for qualitative data, and survey findings were tabulated and described.

**Results:**

Nine qualitative studies and 22 surveys were included, mainly in breast and endometrial cancer. Women treated for breast or endometrial cancer and HCPs were mostly supportive of PIFU. Facilitators for PIFU included convenience, control over own health and avoidance of anxiety‐inducing clinic appointments. Barriers included loss of reassurance from scheduled visits and lack of confidence in self‐management. HCPs were supportive of PIFU but concerned about resistance to change, unsuitability of PIFU for some patients and costs.

**Conclusion:**

PIFU is viewed mostly positively by women treated for breast or endometrial cancer, and by HCPs, but further evidence is needed from a wider range of cancers, men, and more representative samples.

A protocol was registered with PROSPERO (CRD42020181412).

## 1 INTRODUCTION

Worldwide there were an estimated 18.1 million people with cancer in 2018, and that figure is expected to almost double by 2040.^1^ Advances in early detection and treatment mean that the number of cancer survivors worldwide is also rising, with approximately 43.8 million cancer survivors in 2018.^2^

Most people will receive long‐term follow‐up (FU) care after cancer to look for signs of recurrence, as early detection is thought to improve survival.^3^ Traditionally, this type of follow‐up involves scheduled visits to a cancer specialist in a hospital setting, which can be expensive for healthcare systems, can be perceived as burdensome by some patients and may not address specific patient needs.^3^ There is a lack of both evidence and consensus around the intensity, setting, duration or type of follow‐up that should be used in the management of common cancers.^4^ However, current FU models in cancer are increasingly seen to be unsustainable and there is growing interest in alternative FU approaches.^3^, ^5^, ^6^ This has been intensified by the COVID‐19 pandemic, when alternative models of patient FU such as remote or reduced appointments had to be utilised.^7^

Patient‐initiated follow‐up (PIFU) could potentially improve the efficiency of follow‐up by avoiding costs of missed or unnecessary appointments, with comparable clinical outcomes across different types of cancer.^5^, ^8^ It also has the potential to meet the needs of patients in a more flexible and targeted way, for example seeing a specialist sooner than planned FU would have allowed, which in turn could improve patient satisfaction.^9^ Studies in gynaecological cancer patients have found that a majority of patients experience symptomatic recurrence, but many fail to recognise the significance of these symptoms and/or fail to make an appointment earlier than scheduled, suggesting that routine FU can delay the diagnosis of recurrence.^10^, ^11^ UK National Health Service (NHS) guidance on PIFU suggests that PIFU is suitable for oncology, but that a patient's ability to benefit from PIFU needs to be carefully considered; PIFU may not be suitable, for example, for patients with complex needs.^12^ The guidance also highlights the need for safety nets to ensure patients are contacted within specific timeframes if they have not initiated contact themselves.

In PIFU, face‐to‐face hospital appointments are not routinely scheduled, instead patients are given information on signs and symptoms of recurrence and can self‐refer to specialist services on an ‘on‐demand’ basis.^13^ A combination of PIFU and planned FUs can also be offered.^12^ Depending on the type of cancer, this may include some scheduled imaging or other tests (e.g. mammograms for breast cancer or CT scans for colorectal cancer).^14^, ^15^ Where the implementation of PIFU is being considered, it is important to understand whether this approach is acceptable to patients and whether they would be willing and able to use PIFU. Similarly, it is important to gain an understanding of the level of acceptance of PIFU amongst healthcare professionals (HCPs), and whether there are any barriers that would prevent successful implementation. This systematic review aims to draw together all the existing evidence on patient and HCP views, opinions and preferences relating to PIFU in cancer.

## 2 METHODS

A protocol was registered with PROSPERO (CRD42020181412).^16^ Reporting of the systematic review has been informed by ENTREQ guidelines.^17^

### 2.1 Searches

Searches were undertaken in MEDLINE and MEDLINE In‐Process (OVID), Embase (OVID) and CINAHL (EBSCO) from inception to January 2022. Reference lists of relevant reviews and included studies were scanned and experts contacted. There was no restriction by language or publication type. Searches combined text and index terms relating to PIFU; cancer; and patient perspectives, qualitative research, surveys and questionnaires. As the terminology used for PIFU is variable, several alternative terms were used (see Data S5 for sample search strategy).

### 2.2 Study eligibility criteria and screening

**FIGURE 1:**
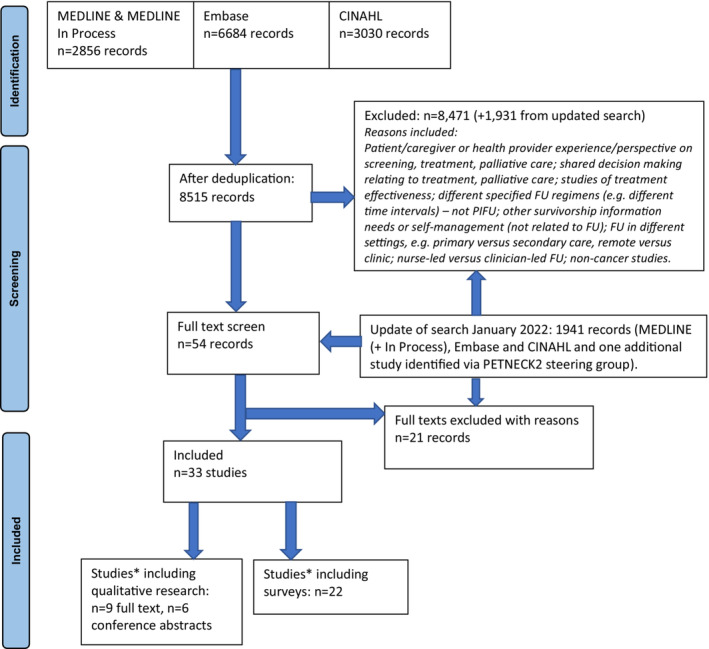
PRISMA flowchart. *4 studies represented in both categories.

Two reviewers independently screened titles and abstracts, or full texts where necessary, using predefined screening criteria (see Table 1). Disagreements were resolved through discussion. Covidence systematic review software (Veritas Health Innovation, Melbourne, Australia) and Rayyan software was used to screen and record decisions.^18^ The study selection process for all studies is shown in Figure 1 (see Data S6 for reasons for exclusion).

**TABLE 1:** Study eligibility criteria.

	Inclusion	Exclusion
Population	Adult (≥18 years) cancer survivors who had completed curatively intended cancer treatment with experience of PIFU or expressing a view on PIFU. Carers/family members of such cancer survivors. HCPs with experience of PIFU or expressing a view on PIFU. Any type of cancer.	Patients with active disease undergoing treatment. Children.
Follow‐up strategy	Any type of FU strategy for recurrence (first or subsequent) providing it includes a form of PIFU. PIFU as the only or main component of a follow‐up strategy, or as an adjunct to standard follow‐up.	Any other follow‐up models that do not include an element of PIFU.
Study design	Qualitative studies, or the qualitative component from mixed methods studies, with a focus on follow‐up strategies and which provide data on PIFU. No restrictions on setting or type of data collection No restriction on reporting, e.g. full report or conference abstract only. Quantitative surveys eliciting views on acceptability and/or preferences related to PIFU.	Studies with no primary data and single case reports.
Outcomes	Patients' (or carers'/family members' or HCP's) views, opinions, experiences, behaviours and preferences relating to PIFU.	Effectiveness or cost‐effectiveness of PIFU.

### 2.3 Data extraction and quality assessment

Data were extracted by one reviewer using a predesigned and piloted data extraction form and checked by a second. Disagreements were resolved through discussion. Quality assessment of qualitative studies was based on the CASP Qualitative Research Checklist.^19^ For surveys, details on questionnaire design, sampling method, response rate and representativeness of sample were noted.

### 2.4 Analysis

All qualitative data in the form of author‐reported concepts/themes relevant to PIFU were extracted by one reviewer (JD) and checked by a second (DM). Patient or HCP quotes were extracted where these covered additional concepts. Article findings (relevant to PIFU only) were independently coded line‐by‐line, with ongoing discussion of codes and levels of coding. Codes were derived from the data. Coding was then organised into related areas to construct overarching descriptive themes. Data were grouped according to whether they were supportive of PIFU (facilitators), or unsupportive of PIFU (barriers). Any similarities between patient and HCP themes were noted. Only qualitative data were considered from mixed methods studies. Quantitative survey findings relating to PIFU were grouped by different types of cancer and described narratively, with main results tabulated. Quantitative synthesis (e.g. of proportions of responses to questionnaire items) was not possible due to substantial variability between studies in population (e.g. type of cancer), type of questions/questionnaires or type of hypothetical FU scenarios that were provided.

## 3 RESULTS—QUALITATIVE STUDIES

### 3.1 Volume of evidence

Nine studies containing qualitative data were included: three qualitative interview studies,^6^, ^20^, ^21^ four mixed methods studies that included interviews,^13^, ^15^, ^22^, ^23^ and two surveys that included a limited amount of qualitative data from a ‘free text’ section.^24^, ^25^ The findings of a further six qualitative studies that were reported as conference abstracts only and contained limited information are described in the Data S4 (conference abstract findings) and not further considered here.^26^, ^27^, ^28^, ^29^, ^30^, ^31^

### 3.2 Study characteristics

Seven of the nine qualitative studies reported patients' views and two on HCPs'. Eight studies were from the UK and one from Sweden (Table 2: main qualitative study characteristics). Four studies reported breast cancer patients' views on PIFU.^15^, ^20^, ^23^, ^24^ In three of these studies (*n* = 30^15^; *n* = 19^20^; *n* = 20^23^), patients with experience of PIFU took part in qualitative interviews. In these studies, PIFU meant women had open access to appointments as needed and received a yearly mammogram, but had no other routine clinic appointments. The fourth study was a survey that included ‘free text’ comments of women in routine FU asking about preferences for future FU (including PIFU).^24^ It was unclear how many participants contributed to qualitative data in this study (this study is also included in the section on surveys).

**TABLE 2:** Main qualitative study characteristics.

Author, year, country	Type and focus of study	Recruitment of participants	(a) setting and method for data collection; (b) person collecting data; (c) type of analysis	(a) number of participants; (b) mean (SD) age; (c) ethnicity; (d) HCP role (if applicable)	(a) type of cancer; (b) time since treatment end; (c) length of participating in PIFU	Description of PIFU	Representativeness of sample
Breast cancer							
Brown 2002, UK^15^	Randomised controlled trial, but included structured interviews Comparison of standard clinical follow‐up with PIFU in women treated for breast cancer	All women in the randomised controlled trial	(a) not stated; some interviews over the telephone. Structured 5–10‐min interviews; items were influenced by relevant literature and from results of a pilot study investigating the attitudes of 100 women to their outpatient follow‐up at the same clinics conducted by the authors (b) research nurse (c) content analysis with each topic reported coded as 1 (no) and 2 (yes). Frequency data then collected for each group	(a) 27/30 at 6 months, 28/30 at 1 year (PIFU group), 24/31 at 6 months, 26/31 at 1 year (standard clinic group) (b) Mean age 68 (53–87) PIFU group, 63 (48–83) standard clinic group. (c) no details	(a) stage I breast cancer (b) at least 1 year and up to 5 years since treatment (c) 1 year of PIFU	Women given written information on the signs and symptoms of recurrence and instructed to telephone the Breast Care Nurse if they encountered any problems. All women also had a yearly mammogram.	50% of those approached refused to participate in study.
Koinberg 2002, Sweden^20^	Qualitative. Breast cancer patients' satisfaction with a spontaneous system of check‐ups.	Strategic sample of women who had participated in the specialist nurse intervention.	(a) University hospital; semistructured interviews (20–40 min); questions previously tested in pilot interviews (b) principle author (oncology nurse) (c) qualitative descriptive design with phenomenographic approach	(a) 19 participants. (b) mean age 63 (no SD) (c) no details	(a) stage I or II breast cancer (b) between 2 and 7 years after surgery (c) length of PIFU unclear	Women could contact a specialist nurse when the need arose. No details on whether a symptom checklist was provided. All women also had a yearly mammogram.	Strategically chosen sample to get as wide a variation as possible (in age, education, marital status, year since operation). No details on patient characteristics.
Moore & Matheson 2022, UK^23^	Qualitative study (as part of wider mixed methods study). Experience of a ‘Supported Early Discharge Follow‐up’ (PIFU) service.	Purposive subsample from a larger group of patients taking part in PIFU at two UK hospitals.	(a) Semistructured interviews via telephone (audio‐recorded), lasting 30–45 min (range 25–60 min) and using an interview topic guide (b) Interviews conducted by an experienced health researcher not involved with patient's clinical care (c) Thematic analysis	(a) 20 participants (b) 10% under 40 years, 35% 40–55 years, 55% over 55 years (c) 1 (5%) White British and Caribbean mixed race, 19 (95%) White British	(a) any primary breast cancer except those on endocrine therapy only or with secondary/metastatic or locally advanced disease. (b) around 3 months after treatment end in one centre and 6 months in the other centre (up to 12 months for some patients due to staffing issues in the latter). (c) women were on average 2–3 years (postdiagnosis) at time of interview	Holistic needs assessment and End of Treatment Summary through nurse‐led clinics followed by discharge from hospital around 6 months following the end of adjuvant treatment; open access to appointments (within 2 weeks); annual mammography for 5 years.	Purposive sampling to ensure that interviewees included a range of ages, breast cancer treatments and levels of needs and concerns (as reported via survey). Patients were excluded from PIFU if they had: learning difficulties; mental health issues; endocrine therapy only; secondary or metastatic or locally advanced disease; recruited on clinical trials.
Muktar 2015, UK^24^	Survey with ‘free text’ comments section *(NB only qualitative data considered here)* Breast cancer patients' (in standard FU) preferences for future FU including open‐access FU.	All eligible patients at one hospital who had received at least 6 months of standard FU were invited to complete a questionnaire during a 6‐month period.	(a) teaching hospital; ‘free text’ section on questionnaire (b) anonymised questionnaire (c) ‘key themes identified’	(a) 317 women recruited (unclear how many invited). Unclear how many patients contributed to qualitative data (b) age not stated (c) 78% Caucasian, 9% Afro‐Caribbean, 7% Indo‐Asian	(a) any stage of breast cancer (b) at least 6 months (c) N/A (all in routine FU)	Hypothetical open‐access FU scenario (flowchart) presented as part of the questionnaire.	Unclear how many patients contributed to qualitative data from wider pool of those responding to survey.
Endometrial cancer							
Beaver 2020, UK^13^	Mixed methods study. Acceptability and feasibility of patient‐initiated follow‐up for women treated for stage I endometrial cancer.	Gynaecology oncologists and clinical nurse specialists asked to identify suitable patients from outpatient clinics; those indicating interest discussed the study with a researcher.	(a) setting not stated; semistructured interviews. (b) no detail on who was collecting data (c) content/thematic analysis	(a) of 65 women eligible for study, 17 agreed to participate; 14/17 participated in interviews (b) mean age 59.41 (10.82) (c) 16 White, 1 Indian	(a) stage I endometrial (b) mean 6 months (c) median 9 months (range 7–10 months)	Patients asked to forego hospital outpatient appointments, supported by a self‐management approach. Information given on signs and symptoms of recurrence and who to contact. Hospital‐based appointment at the end of the study.	26% of eligible patients participated. No data on reasons for nonparticipation or characteristics of nonparticipating patients.
Kumarakulasingam 2019, UK^22^	Mixed methods study. Acceptability of PIFU for endometrial cancer.	Randomly sampled from 4 groups enrolled on the PIFU scheme introduced at University Hospitals Leicester (4 groups: British White; non‐British White; started on PIFU; transferred from hospital FU to PIFU).	(a) outpatients' clinic room; semistructured interviews (20–58 min) (b) two female members of the research team (c) thematic analysis; triangulation with quantitative aspects	(a) 21 women of 51 contacted (b) age not stated for the 21 women (c) 89.5% White British, 10.5% non‐White British (22 British South Asian, 2 African/Afro‐Caribbean)	(a) early stage endometrial PIFU immediately after end of treatment or transferred from hospital FU (b) time in hospital FU not known (c) median 14 months (95% CI 12.9, 14.3 months)	Contact details of clinical nurse specialist provided at end of treatment appointment, as well as written information on signs and symptoms that should prompt medical review. 6‐ and 12‐month telephone calls to ensure patient was happy to continue on PIFU and had contact details.	21/51 (41%) contacted agreed to interviews. No details on reasons for non‐participation or characteristics of nonparticipating patients.
Sharma 2020, UK^25^	Survey with ‘free text’ comments section *(NB only qualitative data considered here)* Patient satisfaction with PIFU for endometrial cancer	All women who underwent surgery for stage I endometrial cancer in a district general hospital 2013–2018); all had agreed to PIFU.	(a) data collected via a telephone call where patients were asked a series of prespecified questions. One was an open‐ended question asking for any comments. (b) clinical nurse specialist (c) no details on type of analysis *(‘individual comments were noted*’).	(a) 94/104 (90%) responded to the survey but unclear how many provided free text comments. Not all women were contacted each year. (b) no details (c) no details	(a) stage I endometrial cancer (b) PIFU commenced 2–4 weeks after surgery (c) between 2 and 5 years	Patient‐led telephone follow‐up, where they could call the clinical nurse specialist team at any time if they had any concerns.	Unclear as not known how many/which women provided free text comments.
Head and neck cancer							
Lorenc 2022, UK^32^	Qualitative study. Clinicians' views of patient‐initiated follow‐up in head and neck cancer to inform the design of a trial on PIFU compared with routine FU.	Via personal contacts of the team and multidisciplinary professional body mailing lists representing HNC clinicians. Some participants suggested colleagues.	(a) eight online focus groups with between one and six participants with the same role (b) the facilitators did not know the participants beforehand (c) thematic analysis	(a) 34 participants. (b) no details (c) no details (d) ear, nose and throat and maxillofacial surgeons, oncologists, clinical nurse specialists, allied health professionals (speech and language therapists, dietitians and radiographers).	(a) head and neck (b) N/A (c) None of the participants had direct experience of PIFU. PIFU described is that of planned trial	*NB planned PIFU for future trial* Routine FU for first‐year post‐treatment. PET‐CT scan at study entry followed by PIFU (if scan negative). PIFU includes an allied health professional (AHP)/nurse‐led education session, an information and support resource and rapid access to urgent clinical appointments within 2 weeks. The information and support resource provides information on symptoms to be aware of, a diary to monitor symptoms and contact details for easy access to clinical team.	Range of roles (*n* = 6) included and participants from various geographical regions. Many participants were colleagues of those involved in an upcoming trial of PIFU compared with routine FU and/or had been involved in the grant application for the trial.
Any cancer							
Williamson 2020, UK^6^	Qualitative. Healthcare professionals' views of alternative strategies for follow‐up care	Combination of convenience and snowball sampling to identify range of participants.	(a) semistructured interview by telephone or face‐to‐face if preferred (b) interview conducted by one researcher unknown to the participants (c) thematic analysis.	(a) 21 participants time in current post mean of 7 years (range 1.5–18 years). (b) no details (c) no details (d) clinical nurse specialists, lead cancer nurses, consultant surgeons, oncologists, GPs, commissioners of cancer services and NHS managers	(a) any type of cancer (some focus on breast and prostate) (b) N/A (c) length of experience with PIFU variable and/or limited given that programmes were in early phases of implementation.	Not one particular type. Views sought on various follow‐up strategies. All participants reported that their institutions had attempted to implement alternative FU models of care; most were in the developmental or early phases of implementation. Focus on common cancers such as breast and prostate.	Range of roles (*n* = 12) included and participants from various geographical regions. But small number of participants given the number of different types of post/roles.

Three studies reported on endometrial cancer: two mixed methods studies which included qualitative interviews (*n* = 14^13^; *n* = 21^22^) and a telephone survey^25^ with the opportunity for additional comments (unclear how many participants contributed to qualitative data). In each study, all women had direct experience of PIFU through the provision of information on signs and symptoms of recurrence and the opportunity to contact specialist nurses who could instigate referrals. There were no routine clinic visits. In most studies, participants were unlikely to be representative of a wider cancer population as they: excluded women with mental health issues^15^, ^22^; included women who were mainly white, well educated, and younger than average^13^; were at low risk of recurrence^15^, ^22^; and included only those who had consented to take part in PIFU and/or interviews. One study included both British White and non‐British White participants in order to reflect the diverse background of local participants.^22^

The views of HCPs on PIFU were reported in two studies from the UK: one study (*n* = 43) focussed on head and neck cancer (*n* = 43)^21^ and the other on any cancer (*n* = 21).^6^ Participants included surgeons, oncologists, nurse specialists in both studies and additionally allied health professionals in one study^21^ and commissioners and managers in the other.^6^ Participants had either no direct experience,^21^ or limited/variable experience of PIFU.^6^ In the head and neck cancer study, some participants were personal contacts of those planning a trial of PIFU and as such may have been biased favourably towards PIFU.^21^ The number of participants in the study on any cancer is likely to have been too small given the breadth of the question (any cancer, any FU mode, various HCP roles), and the representativeness is uncertain.

### 3.3 Quality of evidence

Studies were of overall good methodological quality, with the exception of two studies that contained only a limited amount of qualitative data (as part of ‘free text’ section of surveys) and reported few details on analysis methods.^24^, ^25^ Some studies did not fully report on the researchers' own role in influencing the analysis, the relationship between researchers and participants, or details of the interview process and analysis (see Data S2 for quality assessment of qualitative studies).

### 3.4 Qualitative study findings

Findings are summarised below across five themes and key findings, and illustrative quotes are presented in Table 3.

**TABLE 3:** Illustrative quotes.

Quotes in support of PIFU or concerns around routine FU	Key findings
*Patient: ‘I am very, very anxious when I am coming [to hospital follow‐up] and probably for a couple of days before’*.^13^ *Patient: ‘It becomes a bit of a pain coming in every 4 months, every 6 months, when actually there's not anything wrong with you and it's a waste of your time, bus money, petrol money, whatever the consultant's time, when there's actually nothing wrong with you’*.^22^ *Patient: ‘I get upset looking at the leaflets—will call if anything worrying. Coming to hospital would bring it all back and I would rather not think about it’*.^25^ *Surgeon: ‘It's prescriptive and certainly not evidence‐based. it's a little bit archaic, and I think, for a long time, many of my colleagues have felt that we could look at a more sensible way of following up patients, and certainly more evidence‐based’*.^32^ *Oncologist: ‘I think we're probably all in agreement that there is room for improvement in the way that we see the patients on their follow‐up protocol. It sounds like we've all got a very similar, traditional one‐size‐fits all approach to our follow‐up’*.^32^ *Lead cancer nurse: ‘We do have patients who come back who had symptoms weeks ago but thought oh it's alright I've got an appointment coming up’*.^6^	Patients associate routine FU with anxiety and inconvenience, and HCPs see the system as inflexible and outdated.
*Patient: ‘I feel that it's been good that I could phone the same nurse and talk to her and if I was specially worried, like in the beginning, then she arranged an appointment with the doctor so it went very smoothly, I think’*.^20^ *Patient: ‘The nurses were brilliant. I had 45 minutes longer than I would with a doctor so it was good as they could explain everything in detail’*.^25^ *Patient: ‘I used to dread going and I do not dread it any more*.. *. not because I was worried about what the outcome might be, it was a heck of a journey from here to Southampton and the waiting around* etc.’^15^ *Patient: ‘Well I think it gives you confidence, oh what is the word I am looking for, peace of mind you know, that they* [telephone access to breast cancer nurse] *are still keeping an eye on you’*.^15^ *Patient: “I will go back if I need to, I think the top and bottom of it is, if people have got a phone number to ring, they are more confident, aren't they? Like I have got [name of specialist nurse], it is just there if you need it’*.^13^	Patients experience PIFU positively and feel supported by it.
*Patient: ‘I kind of go in there and I feel like it's a bit of a waste of their time and my time. If I had symptoms you kind of would call them … if you had any problems you could possibly ring up anyway and say “I don't feel well” so it's basically like when you self‐assess, you are the one that is going to be self‐assessing anyway aren't you’?* ^13^ *Patient: ‘It stops me having to worry about “I've got an appointment here to come and see this person.” I'm looking out for my own symptoms and know that if I ring up the secretary or the clinic and say “I have this issue, can I come and see somebody?” I can come in. I don't have to go* via *the GP is what I'm saying’*.^22^ *Patient: ‘In terms of [PIFU], it will suit me down to the ground, in that I kind of want to, as best as possible, move on from it, and this allows me just to actually pay attention to my body and if something's wrong I flag it up, whereas I think if I had to wait once a year for my check‐up I would just wait for my check‐up if I thought something was wrong …. It just forces you to take responsibility for your health a little bit and pay attention a bit more. I just wanted to get it done and dusted and out of there. I don't want to be followed up really. I don't want to be reminded that it happened’*.^23^	Patients view taking control of managing their own follow‐up as positive.
*Surgeon: ‘Patients who've been able to quit smoking or alcohol use, or semi reduce it significantly, might be at low risk of recurrence, and perhaps those are patients who could be on a less stringent follow‐up. So, I don't know if you are going to stratify according to risk factors as well’*.^32^	HCPs see the need for tailoring PIFU depending on risk.

Quotes on concerns around PIFU or support for routine FU	
*Patient: ‘I think I would have preferred to come back and seen, physically seen someone… I think it's more just reassurance to meet somebody face to face about it. It's a bit more personal’*.^22^ *Patient:* ‘*I would have liked more appointments with the consultant for reassurance’*.^25^ *Patient: ‘Anyway, I think for a lot of people seeing a doctor gives them confirmation, you're happy to pay the fee’*.^20^ *Clinical nurse specialist: ‘You do always get that group of patients that want to come in and feel reassured just by it, it sounds crazy but just by having the doctor's hands on their neck and things like that they basically feel reassured’*.^32^	Patients and HCPs view the reassurance from routine FU as positive.
*Patient: ‘The only barrier that I think would stop them ringing in is if they worried that it has come back. Because you've got to get your mind around that one first before you go and ring’*.^22^ *Patient: ‘I mean you can examine yourself but you just need somebody to confirm and say yes you haven't found anything or there isn't anything going on there’*.^15^ (Patient in routine FU) *Patient: ‘Prefer not to rely on self‐diagnosis’*.^24^ (Patient in routine FU) *Patient:* ‘*Once you're discharged you don't sort of have any backup for potential reoccurrence, and I almost feel out on a limb. Every lump and bump, you know, you're not trained to say oh, that's a fatty lump, there's no information there to help you. So every time you get a lump and bump you just go into oh my god, here we go …. … it's that void afterwards, that that is my only criticism, because, it's almost like it's a loaded gun and you're waiting for somebody to fire the bullet’*.^23^	Patients worry about relying on self‐assessment for symptoms of recurrence and avoid checking due to fear of recurrence.
*Patient: ‘It would be really handy to have an e‐mail address, or even a number that you could text, not expecting an instant answer … because by the time you've rung two or three times and they've not picked up and you don't really want to leave a message, you get to the point where you think maybe it's not that important, and I won't ring again … So it's more an access issue, in that in your own head you can quite quickly downgrade it if you don't want to be a problem and they are obviously very busy’*.^23^ *Surgeon: ‘A small group of [lower socioeconomic status] patients will say “just do what you think is right.” They don't want to know, you know? I would not trust them, not because I don't like them, it's just that I can't trust them to make a sensible decision to come back if they have a concern’*.^32^ *Consultant nurse: ‘What I'm finding is with the, the older patient is that they struggle with that ownership being put back onto them ‘cos they're used to the paternalistic approach … younger patients seem to accept it better’*.^6^ *Oncologist: ‘[patients may not attend clinic] because they're holding back a problem or they're scared. And it's really how those things get identified, because this potentially can be the way that people keep a problem [hidden] that we would have seen by looking in the whites of their eyes’*.^32^	Patients and HCPs have concerns around access to PIFU.
*Clinical nurse specialist: ‘I think the main concern was if it [patient‐initiated follow‐up] would add to the workload’*.^32^ *Surgeon: ‘There will be some people [clinicians], I think, that the way they approach risk, or just their attitude, they may just say, ‘Well, no, I'm not willing to engage in that [patient‐initiated follow‐up]’*.^32^ *Survivorship Network Manager: ‘… you need to manage patients expectations around that being the sort of follow‐up that they can expect … if you're not careful and you follow up people up at sort of arm's length in that way, it might actually exacerbate the problem of people feeling isolated rather than improve it’*.^6^	HCPs have concerns around change and implementation of PIFU.

#### 3.4.1 Perception of routine FU

Both patients and HCPs thought routine clinic appointments could cause anxiety in some patients.^6^, ^13^, ^21^, ^22^, ^25^ Clinic appointments were associated with an increased fear of recurrence,^13^ painful reminders of the cancer (treatment),^13^, ^23^ and a sign of ‘active’ disease rather than surveillance.^22^ Some patients questioned the value of scheduled visits where risk was low,^22^ or when there were no symptoms.^25^ Current systems were viewed by HCPs as ‘rigid’, ‘unresponsive’, ‘paternalistic’^21^ as well as ‘not patient friendly’, giving ‘artificial support’ and not addressing long‐term effects or patient needs.^6^, ^21^ Patients were worried about wasting health professionals' time if there were no apparent problems^13^, ^22^ and where risk of recurrence was low.^22^

Routine (clinic) FU was however also viewed as reassuring by patients and HCPs,^6^, ^13^, ^15^, ^21^, ^22^, ^24^, ^25^ especially in early stages of FU.^13^, ^22^ Some patients (in PIFU) were anxious about not seeing a doctor^20^ and reported initial difficulties in adjusting to a lack of appointments.^23^ Others were more supportive of PIFU starting after a period of routine FU in the early/acute stages.^13^, ^22^, ^24^ A view amongst HCPs was that the traditional FU model was associated with ‘trust’^6^ and that a lack of routine FU might impede recurrence or metastasis detection (in head and neck cancer).^21^

#### 3.4.2 Access to, and use of, PIFU

Patients thought PIFU was more convenient, for example in terms of travel, cost and waiting times.^13^, ^15^ They valued quick and easy access to (specialist) nurses, who could make onward referrals if necessary,^20^, ^22^, ^25^ and were confident that their concerns would be addressed, particularly where they had already had a positive experience with PIFU.^23^ Contact with a health professional known to the patient was preferred.^20^, ^23^ British South Asian women commented on the value of a Gujarati/Hindi‐speaking nurse.^22^ Reasons for not accessing PIFU included fear of wasting health professionals' time^22^, ^23^ and perceiving GPs as more accessible,^23^ while some women noted a dislike of leaving answerphone messages and difficulties in getting a response via the designated helpline.^23^ The role of PIFU was queried by some, in terms of accessing support not only for symptoms of recurrence but also for ongoing treatment‐related side effects (particularly adjuvant endocrine therapy in breast cancer) and psychological issues, including fear of recurrence, which were perceived as unmet needs.^23^ HCPs stressed that a route to urgent appointment or specialist care was important and that this needed to be clear, efficient, reliable and quick, with designated points of contact.^6^, ^21^

#### 3.4.3 Patient self‐management and recognising recurrence

Patients on PIFU liked having more control over their own health and making their own decisions^13^, ^22^, ^23^ and were confident they would recognise signs and symptoms of recurrence providing they had received detailed information.^13^ One study found that greater emotional well‐being on PIFU was influenced by personality (e.g. being optimistic), good social support and coping strategies, and sufficient financial resources.^23^ HCPs were also in favour of PIFU giving patients more control to enable them to take more responsibility for their own health, including looking for signs of recurrence.^21^ Patients and HCPs were however also concerned that patients would not recognise symptoms, would ignore symptoms, or avoid self‐examination due to fear of recurrence.^13^, ^15^, ^20^, ^22^, ^23^ Some patients did not feel they had sufficient information (e.g. on breast self‐examination) to prepare them for PIFU.^23^ In one study, participants suggested a (routine) FU appointment after 3 or 5 years of PIFU for additional reassurance.^25^

HCPs thought self‐management approaches may not be suitable for elderly patients, patients with mental or physical health issues or who were otherwise vulnerable,^6^, ^21^ and one study found poorer emotional well‐being on PIFU where patients had existing physical or mental co‐morbidities or had other life stressors.^23^

#### 3.4.4 Tailoring PIFU to underlying risk of recurrence

HCPs felt that PIFU would be less suitable for patients with complex needs, rare forms of cancer or poorer prognosis/high risk of recurrence and that the suitability of PIFU would vary depending on (sub‐)type of cancer.^6^, ^21^ Prostate cancer and cancers with obvious signs and symptoms were seen as more suitable than ovarian cancer, for example.^6^ Head and neck cancer HCPs noted that patients less likely to engage with PIFU may also be those at higher risk of recurrence, which may result in worse health outcomes.^21^

#### 3.4.5 Change and implementation of PIFU

There was support for changes to routine FU amongst head and neck cancer HCPs, while acknowledging that some colleagues may be more risk averse and reluctant to change.^21^ Changing a ‘cultural’ view of both patients and HCPs in terms of FU was seen as potentially difficult.^6^ A lack of evidence on effectiveness for either routine FU^21^ or PIFU^6^ was also mentioned. Managing patients' expectations regarding FU was considered important.^6^, ^21^

HCPs thought that current FU systems were unsustainable and placed too high a burden on health service (UK‐NHS) resources.^6^, ^21^ However, there was also concern that there was little incentive for (UK‐NHS) hospitals to give up routine FU as they would lose payment for this.^6^ It was also mentioned that funding would still be required for alternative approaches and that funding could not simply be cut,^6^ and there was concern around staffing and potential additional nursing workload.^21^ One study noted that a service specification was viewed as a useful lever when implementing new models, and the importance of communication with commissioners was emphasised.^6^

## 4 RESULTS—SURVEYS

### 4.1 Volume of evidence

Twenty‐two relevant studies containing surveys were identified. Four of these also contained qualitative data, which is included in the qualitative evidence section.^13^, ^22^, ^24^, ^25^

### 4.2 Survey characteristics

*S*urveys were in breast (*n* = 9), head and neck (*n* = 5), endometrial (*n* = 4), breast or gynaecological (*n* = 1), colorectal (*n* = 2) or any cancer (*n* = 1). Most were from the UK (*n* = 16), the remainder from Denmark, Sweden, Italy, Slovenia, Italy and Canada (see Data S3 for survey characteristics and findings). There was variability in type of study (e.g. survey only, audit of existing services, randomised controlled trial) and types of questions posed or (hypothetical) scenarios presented. Only one survey included HCP views in addition to patients' views.^4^ At least some patients in half the surveys (*n* = 12, 55%) had direct experience of PIFU, this included breast, endometrial and colorectal cancer patients. PIFU entailed the provision of information on signs and symptoms of recurrence and a mechanism for patients to contact HCPs and/or self‐refer if there were concerns. For breast and colorectal cancer, it also included scheduled mammograms or CT scans and colonoscopies, respectively. In the other surveys, participants were given hypothetical questions or scenarios on PIFU.

### 4.3 Quality of surveys

There was generally a lack of detail on how questionnaires were developed or whether they were validated. Sampling strategies appeared mostly satisfactory, but some studies reported that their sample was unlikely to be representative of a wider cancer population.^13^, ^24^, ^33^, ^34^, ^35^, ^36^, ^37^, ^38^, ^39^ This could be due to areas or centres participants were recruited from, eligibility for a wider study (e.g. RCT) or self‐selection bias. Response rates, where reported, ranged between 60% and 90% (see Data S3 for survey characteristics and findings).

### 4.4 Survey findings

#### 4.4.1 Breast cancer

Six surveys based on patients' experience of PIFU (with regular mammograms) found that most patients (88%–100%) were generally as satisfied with open‐access/PIFU systems as they were with routine FU or that the systems were comparable in terms of addressing concerns.^38^, ^40^, ^41^, ^42^, ^43^ Three surveys^24^, ^44^, ^45^ of women in routine FU found that the majority (90% where reported) were satisfied with this FU and wished to continue with it; one found that around half would be willing to be discharged from hospital FU after 3 years if an open‐access system was in place.^44^

#### 4.4.2 Endometrial cancer

Three surveys were of women who had participated in PIFU.^13^, ^22^, ^25^ Three‐fifths (59%–63%; 2 studies) of patients indicated support for a system of early hospital discharge/PIFU^13^, ^22^ or ‘most’ were satisfied with the service, but this related to treatment as well as FU (one study).^25^ Patients who had been in a trial of hospital versus telephone FU were asked about future FU preferences.^33^ Depending on trial arm, open‐access PIFU was ranked 4th or 6th amongst eight scenarios and was less popular than hospital appointments with a doctor and/or a specialist nurse (ranked 1st to 3rd).

#### 4.4.3 Breast or gynaecological cancer

One survey included women with either breast or gynaecological cancer as well as HCPs, none of whom had direct experience of PIFU.^46^ Most respondents were in favour of regular appointments in terms of making patients feel ‘safe’ (92% patients, 65% HCP). Only around 26% of patients and 12% of HCPs thought patients would prefer symptom‐led appointments.

#### 4.4.4 Head and neck cancer

Three surveys^34^, ^37^, ^47^ found that the majority (80–89%) of patients preferred routine or scheduled FU when asked to consider PIFU as a hypothetical alternative, and one survey found that patients were in favour of a less intensive, more patient‐led FU approach.^48^ A further study presented a range of hypothetical FU scenarios consisting of more or less frequent FU, with regular or symptom‐prompted imaging.^36^ The most preferred scenario was hospital‐based FU with frequency of visits decreasing over time and routinely scheduled imaging irrespective of individual risk of recurrence. No patients had direct experience of PIFU.

#### 4.4.5 Colorectal cancer

Two studies^14^, ^39^ found that most patients (97% and 73%) with experience of PIFU found this to be acceptable, and in one study, similar to routine FU in terms of how expectations were met.^14^ Patients in both studies had regular scheduled scans in addition to PIFU.

#### 4.4.6 Any cancer

One survey asked patients, carers and HCPs about experiences of and preferences for PIFU.^4^ Of those with experience of PIFU (27% of patients; 37% of HCPs), 80% expressed a preference for it. No results were presented for PIFU preferences amongst those with no direct experience of it.

## 5 DISCUSSION

PIFU appears to be positively viewed by a majority of patients treated for breast cancer, and, to a slightly lesser degree, patients treated for endometrial cancer, provided reliable systems are in place to ensure easy access to specialists. A smaller proportion of patients view PIFU as less acceptable or suitable; this proportion is likely to be higher in a ‘real life’ setting when study inclusion criteria and/or self‐selection do not apply. Barriers to PIFU included a loss of reassurance from regular follow‐up, difficulties accessing PIFU, or avoidance or fear of self‐examination. PIFU may also be more difficult to access by non‐English language speakers unless support is in place. HCPs noted that there are some patient groups for whom PIFU may not be suitable including those with complex needs or mental and physical health issues, most of which are likely to have been excluded from existing studies.

A very limited amount of evidence (based on two surveys) suggests a potential role for PIFU in colorectal cancer. Based on survey data, patients were as satisfied or more satisfied with PIFU where they had direct experience of it (in breast, endometrial and colorectal cancer), and patients given hypothetical scenarios of PIFU were more likely to state a preference for continuing with routine FU (in breast, gynaecological, endometrial and head and neck cancer). This may reflect positive experiences with routine FU already received, or a reluctance to change from what has worked so far. Conversely, satisfaction with PIFU may also reflect patient recruitment to PIFU studies; where participation in a PIFU scheme is dependent on patient consent, then these patients may be favourably inclined to PIFU. The participation in a study in itself may have an effect on how survey questions are answered, and could, for example, depend on knowledge of other FU options (e.g. if there is a control group); the method and frequency of obtaining data (e.g. if collected by someone involved in the study); or the attention given by health professionals as a result of knowledge (and beliefs) around the different FU options.^49^

Acceptability of PIFU may be influenced by provision of scheduled imaging or other tests to support PIFU, as in the breast and colorectal cancer studies. All types of PIFU in breast cancer studies included regular mammograms even though scheduled clinic/hospital FU visits were replaced by ‘on‐demand’ visits. The studies on PIFU in colorectal cancer also included some scheduled imaging or other tests. Some versions of ‘PIFU’ may thus not be solely patient‐led but rather be a combination of PIFU and regular tests at the hospital, for example routine scans. This in turn may affect the extent to which PIFU is deemed acceptable to patients and HCPs. For example, in one breast cancer study, some women felt reassured by regular mammograms.^20^ However, scans are also known to cause anxiety during the time leading up to the scan and the time spent waiting for results, and feelings of reassurance may not be sustained.^50^

Based on limited evidence, HCPs are generally supportive of PIFU, but have concerns about managing patient and HCP expectations, and about patients who have difficulties engaging with PIFU. HCPs note that barriers to PIFU include patients' communication or language difficulties as well as a lack of technological developments to aid patient–clinician communication.^51^ Some studies have shown that patients may not request urgent appointments despite recognising symptoms^52^, ^53^ and that regular FU can facilitate access to specialists or tests in these cases.^54^, ^55^

The reassurance regular FU can provide needs to be weighed against increased anxiety, the inconvenience of potentially unnecessary hospital visits and the fact that reassurance is often only temporary.^15^, ^22^, ^55^ Further, some hospital‐based follow‐up may not sufficiently address patient needs; the ENDCAT trial in endometrial cancer found that patients were more satisfied with some aspects of nurse‐led telephone FU appointments than they were with doctor‐led hospital FU appointments.^56^

There is some concern that regular FU can delay patient presentation when they feel symptoms have changed and so delay recurrence identification. Participants in the studies commenced PIFU at varying times after treatment completion and time in routine FU. One study in breast cancer patients suggested that emotional and information needs are greater in the immediate post‐treatment phase and that these might be initially better dealt with at a clinic.^15^ Some patients may therefore be more favourably inclined to transition to PIFU after a period of routine, clinic‐based FU.

PIFU was not seen as suitable for all patients or types of cancer and may have to be implemented in a risk‐stratified way, for example taking into account likelihood of recurrence, and ability of patients to recognise recurrence.^6^, ^21^ In practice, patients are sometimes enrolled into PIFU on the basis of risk, for example patients with low‐risk endometrial^13^ or low‐risk breast cancer.^15^ Fear of recurrence was noted by some study participants. This is common in cancer patients, and one study in endometrial cancer found that routine FU decreased fear of recurrence significantly more than PIFU, though the difference was small,^57^ while another in breast cancer found a slightly higher, but not statistically significant, level of fear with PIFU compared with regular FU.^58^

Any findings on preferences related to PIFU need to be considered in the context of effectiveness. There is currently insufficient evidence on the impact of PIFU on long‐term outcomes such as recurrence or mortality compared with routine follow‐up.^59^ The value of detecting an asymptomatic recurrence with scheduled FU will likely vary depending on type of cancer and available treatment options; some evidence suggests a survival benefit from asymptomatic recurrence detection based on imaging or other diagnostic tests for gastric cancer,^60^ breast cancer,^61^ bladder cancer^62^ and colon cancer.^63^ A study in ovarian cancer found no evidence of a survival benefit with early treatment based on raised CA125 levels compared with delayed treatment based on symptoms.^64^ A recent systematic review by Kershaw et al. found that PIFU did not have a negative impact on the detection of recurrence in gynaecological cancers but that the psychological impact was conflicting.^9^ There is also uncertainty around whether patient‐initiated appointment systems specifically lead to reduced service utilisation or costs in chronic disease, including cancer.^5^ A study in endometrial cancer has suggested that use of PIFU can lead to cost savings both for the NHS and for the patients, with cases of nonmetastatic recurrence being salvageable.^65^

Strengths of this systematic review include the comprehensive search strategies, which means it is unlikely that evidence has been missed, and the inclusion of both qualitative and quantitative data on preferences. The evidence identified has some limitations. Qualitative studies came from limited geographic and healthcare settings with eight of nine studies being UK based and seven studies exploring patient perspective covering only breast and endometrial cancer. Most of the surveys were also UK based. The substantial interest in PIFU in the UK may be being driven by current NHS priorities, which include supporting providers to implement PIFU.^66^ Samples in the two studies exploring HCP views are also unlikely to have included all relevant specialities and roles.

Resistance to change amongst colleagues and services is a known barrier for the implementation of PIFU, though restrictions during the COVID‐19 pandemic may have accelerated opportunities for change (such as mainly telephone/virtual consultations and reduced appointment frequency) as well as boosting clinician, service and patient enthusiasm for change.^21^, ^67^ While there is evidence that supported self‐management can improve clinical, psychosocial and economic outcomes, there is also a lack of evidence on what optimal self‐management strategies are, and to what extent support by health professionals is needed to make such approaches effective and sustainable.^68^, ^69^ There is health professional support for survivorship courses, but many clinicians report that these are not available for their patients.^51^

Future research on the perception of PIFU should include studies in a wider range of cancers, including men, and patients with varying underlying risk of recurrence, and should consider PIFU in the context of scheduled imaging or other tests. Ideally, such research will be in the context of participants with first‐hand experience of PIFU, as there is evidence to suggest that views differ depending on whether participants have experienced PIFU or are being asked to consider hypothetical scenarios. Such studies should make efforts to engage participants from diverse backgrounds and with a broad range of experiences.

## AUTHOR CONTRIBUTIONS

**Janine Dretzke:** Formal analysis (lead); investigation (lead); methodology (equal); project administration (lead); validation (equal); writing – original draft (lead); writing – review and editing (equal). **Ava Lorenc:** Investigation (equal); validation (equal); writing – original draft (equal); writing – review and editing (equal). **Ada Adriano:** Investigation (supporting); writing – review and editing (equal). **Clare Herd:** Investigation (supporting); writing – review and editing (equal). **Hisham Mehanna:** Conceptualization (equal); funding acquisition (lead); methodology (equal); supervision (equal); writing – review and editing (equal). **Paul Nankivell:** Conceptualization (equal); funding acquisition (equal); methodology (equal); supervision (equal); writing – review and editing (equal). **David J Moore:** Formal analysis (equal); investigation (equal); methodology (equal); validation (equal); writing – original draft (equal); writing – review and editing (equal).

## FUNDING INFORMATION

This work was funded by a National Institute for Health Research (NIHR) Programme Grant for Applied Research (NIHR200861).

## CONFLICT OF INTEREST STATEMENT

Three of the authors (AL, HM and PN) are authors of one of the studies included in this systematic review. None of the other authors report any conflicts of interest.

## Supporting information


Data S1.

Data S2.

Data S3.

Data S4.

Data S5.

Data S6.

Data S7.

## Data Availability

Extracted data from published articles available in supplementary material. All published articles are in the public domain.
