# Supplementary material for: Systematic review of patients’ and healthcare professionals’ views on patient‐initiated follow‐up in treated cancer patients
Source: Cancer Med. 2023 Jun 16;12(15):16531–47. doi: 10.1002/cam4.6243 (PMC10469665; doi:10.1002/cam4.6243)
Supplement: Supplementary file 1 — Data S1. [file CAM4-12-16531-s005.docx]

**Members of the PETNECK2 Research Team - in alphabetical order by first name**

Andreas Karwath (Institute of Cancer and Genomic Sciences, University of Birmingham) [A.Karwath@bham.ac.uk](mailto:A.Karwath@bham.ac.uk)

Barry Main (University Hospitals Bristol and Weston NHS Trust) [B.G.Main@bristol.ac.uk](mailto:B.G.Main@bristol.ac.uk)

Charlotte Firth (Cancer Clinical Trials Unit, University of Birmingham) [C.M.Firth@bham.ac.uk](mailto:C.M.Firth@bham.ac.uk)

Claire Gaunt (Cancer Clinical Trials Unit, University of Birmingham) [C.H.Gaunt@bham.ac.uk](mailto:C.H.Gaunt@bham.ac.uk)

Colin Greaves (School of Sport, Exercise and Rehabilitation Sciences, University of Birmingham) [C.J.Greaves@bham.ac.uk](mailto:C.J.Greaves@bham.ac.uk)

David Moore (Institute of Applied Health Research, University of Birmingham) [D.J.MOORE@bham.ac.uk](mailto:D.J.MOORE@bham.ac.uk)

Eila Watson (Oxford School of Nursing and Midwifery, Oxford Brookes University) [ewatson@brookes.ac.uk](mailto:ewatson@brookes.ac.uk)

Georgios Gkoutos (Institute of Cancer and Genomic Sciences, University of Birmingham) [G.Gkoutos@bham.ac.uk](mailto:G.Gkoutos@bham.ac.uk)

Gozde Ozakinci (Health Psychology Research Group, University of Stirling) [Go7@stir.ac.uk](mailto:Go7@stir.ac.uk)

Jane Wolstenholme (Nuffield Department of Population Health, University of Oxford) [jane.wolstenholme@dph.ox.ac.uk](mailto:jane.wolstenholme@dph.ox.ac.uk)

Janine Dretzke (Institute of Applied Health Research, University of Birmingham) [J.Dretzke@bham.ac.uk](mailto:J.Dretzke@bham.ac.uk)

Jo Brett (Department of Midwifery, Community and Public Health, Oxford Brookes University) [jbrett@brookes.ac.uk](mailto:jbrett@brookes.ac.uk)

Joan Duda (School of Sport, Exercise and Rehabilitation Sciences, University of Birmingham) [J.L.DUDA@bham.ac.uk](mailto:J.L.DUDA@bham.ac.uk)

Lauren Matheson (Oxford Institute of Nursing, Midwifery and Allied Health Research, Oxford Brookes University) [l.matheson@brookes.ac.uk](mailto:l.matheson@brookes.ac.uk)

Louise-Rae Cherrill (Cancer Clinical Trials Unit, University of Birmingham) [l.cherrill@bham.ac.uk](mailto:l.cherrill@bham.ac.uk)

Melanie Calvert (Institute of Applied Health Research, University of Birmingham) [M.Calvert@bham.ac.uk](mailto:M.Calvert@bham.ac.uk)

Philip Kiely (University Hospitals Bristol and Weston NHS Foundation Trust) [Philip.Kiely@UHBristol.nhs.uk](mailto:Philip.Kiely@UHBristol.nhs.uk)

Piers Gaunt (Cancer Clinical Trials Unit, University of Birmingham) [P.Gaunt@bham.ac.uk](mailto:P.Gaunt@bham.ac.uk)

Saisakul Chernbumroong (Institute of Cancer and Genomic Sciences, University of Birmingham) [S.Chernbumroong@bham.ac.uk](mailto:S.Chernbumroong@bham.ac.uk)

Saloni Mittal (Institute of Head and Neck Studies and Education, Institute of Cancer and Genomic Sciences, University of Birmingham) [s.mittal.2@bham.ac.uk](mailto:s.mittal.2@bham.ac.uk)

Steve Thomas (Bristol Dental School, University of Bristol) [Steve.Thomas@bristol.ac.uk](mailto:Steve.Thomas@bristol.ac.uk)

Stuart Winter (Nuffield Department of Surgical Sciences, University of Oxford) [Stuart.Winter@ouh.nhs.uk](mailto:Stuart.Winter@ouh.nhs.uk)

Wailup Wong (East and North Hertfordshire NHS Trust, Mount Vernon Cancer Centre) [wailup.wong@nhs.net](mailto:wailup.wong@nhs.net)
