# Supplementary material for: Systematic review of patients’ and healthcare professionals’ views on patient‐initiated follow‐up in treated cancer patients
Source: Cancer Med. 2023 Jun 16;12(15):16531–47. doi: 10.1002/cam4.6243 (PMC10469665; doi:10.1002/cam4.6243)
Supplement: Supplementary file 2 — Data S2. [file CAM4-12-16531-s002.docx]

**Quality assessment: qualitative studies**

| **Study** | **Was the research design appropriate to address the aims of the research?** | **Was the recruitment strategy appropriate to the aims of the research?** | **Was the data collected in a way that addressed the research issue** | **Has the relationship between researcher and participants been adequately considered?** | **Have ethical issues been taken into consideration?** | **Was the data analysis sufficiently rigorous?** | **Is there a clear statement of findings?** | **How valuable is the research?** |
| --- | --- | --- | --- | --- | --- | --- | --- | --- |
| Beaver 2020^1^ | Yes-rationale given for mixed methods approach. | Partially. All patients who could be eligible for PIFU approached, but low uptake and evidence of sample bias. | Yes/partially. Semi-structured interviews using an interview guide. Interviews were audio-recorded and fully transcribed. No details on saturation. | No details. | Yes. Approval sought from ethics committee. Informed verbal and written consent was obtained from all individuals. Patients given detailed information and time to consider participation.  participants included in this study. | Yes/partially. Reliability of the coding was established through independent coding by two researchers. Any discrepancies were resolved through review of the data and discussion until consensus was reached. Measures were undertaken to ensure the rigour of the research process including line-by-line analysis and regular review and de-briefing involving coders. Lack of consideration of researcher role in influencing analysis. Lack of detail on how data were selected for presentation. | Yes. Comprehensive representation of results. Findings from both approaches (qualitative and quantitative) brought together in analysis. Two independent researchers used. | Valuable in terms of: further areas of research identified; representativeness of findings considered; findings set in context of current practice and research in the field. |
| Brown 2002^2^ | Yes -appropriate to answer research question (patient satisfaction) | Yes, but recruitment was difficult with half of those approached refusing to participate. All were at least 1 year post-treatment and highly anxious women were excluded | Yes/partially. 5-10 minute structured interviews using items influenced by relevant literature and results of a pilot study. Audio-recorded with permission. Some interviews over the telephone. Interviews transcribed in full or, if not recorded, field notes were used. No details on saturation. | No details. Presentation bias considered: the research nurse was associated with the clinic (recruitment setting), which may have led to more favourable responses. | Yes. Ethical approval gained. | Yes/partially. Limited description of analysis methods (content analysis).  Contradictory data were  taken into account. | Yes/partially. Detailed presentation of frequencies of responses. No details of whether more than one analyst was involved.  Findings discussed in relation to  original research question. | Valuable in terms of: further areas of research identified; representativeness of findings considered; findings set in context of current practice and research in the field. |
| Koinberg 2002^3^ | Yes -appropriate to answer research question (patient satisfaction) | Partially. Strategic sample to ensure variability of patient characteristics, but no details on characteristics of all PIFU participants. | Yes/partially. Semi-structured interviews using questions identified though pilot interviews. Interviews were audio-recorded and fully transcribed. No details on saturation. | Insufficient detail. Participants not known to nurse conducting interviews. No further details. | Yes. Study approved by ethics committee. Written information on study and explanation that participation was voluntary provided a week before study. | Yes. Detailed description of analysis process, including how themes were derived. Contradictory data highlighted. Each patient had made at least one statement belonging to  each descriptive category.  Assessment performed with both advisors to be aware of possible subjective interpretations by the main interviewer. | Yes. Objective representation of results. Collaboration between three analysts. Findings discussed in relation to original research question. | Valuable in terms of: further areas of research identified; representativeness of findings considered; findings set in context of current practice and research in the field. |
| Kumarakulasingam 2019^4^ | Yes-rationale given for use of mixed methods approach. | Yes/partially. Random sample across groups stratified by ethnicity and mode of entry into PIFU. Representation of non-White British women and non-English speakers. Lack of detail on patient characteristics of those (not) agreeing to interview. | Yes. Semi-structured interviews using an interview guide until saturation was achieved. Interviews were audio-recorded and fully transcribed. | No details. | Yes. Ethical approval granted for patient interviews. Interviews recorded with knowledge and consent of the interviewees. | Yes/partially. Detailed description of analysis process. Two researchers independently coded the data, and similar codes were found to have been applied. All patient experiences were reported in the analysis. Lack of consideration of researcher role in influencing analysis. | Yes. Comprehensive representation of results. Two independent researchers used. Triangulation used to consider findings from qualitative and quantitative aspects and to determine level of agreement. | Valuable in terms of: representativeness of findings considered; findings set in context of current practice and research in the field; transferability of findings to other cancers considered. |
| Lorenc 2021^5^ | Yes - rationale given for qualitative work to inform study design in a new intervention. | Yes/partially. Clinicians recruited from personal contacts and professional body. Volunteers may have been biased towards PIFU/Petneck study as they had already heard of it. | Yes - focus groups useful for exploring views and concerns. Topic guide not presented but topics outlined. | Yes - mentions that they did not know each other. | Yes. Ethical approval granted and patient consent given, and transcripts were anonymous. | Yes/partially. Description of how themes were derived and of analysis process. Two researchers collaborated on analysis. But not clear how quotes were chosen. Lack of consideration of researcher role in influencing analysis. | Yes/partially. Comprehensive representation of results. Two researchers collaborated on analysis. Could be better discussion of the credibility of the findings/evidence for and against the findings. | Valuable as the implications of the work are very clear. Some discussion of transferability. |
| Moore & Matheson 2022^6^ | Yes --appropriate to answer research question (patient experience and views) | Yes/mostly -purposive sample to include a range of ages, breast cancer treatments and levels of needs. Only one participant from a non-white ethnic background reflecting lack of diversity of wider sample. | Yes. Semi-structured interviews using an interview guide Interviews were audio-recorded and fully transcribed. Saturation was achieved for major themes. | Yes - Interviews conducted by a health  researcher not  involved with patient’s clinical care. | Yes-ethical approval granted. Respondents  were assured raw data would remain confidential and would not be shared. | Yes/mostly. Description of how themes were derived and of analysis process. Several transcripts reviewed by two additional researchers and themes discussed at wider team meetings.  But not clear how quotes were chosen. Lack of consideration of researcher role in influencing analysis. | Yes. Comprehensive representation of results.  Relationships between themes explored, as well as convergence and divergence between and within participants. | Valuable in terms of: representativeness of findings considered; findings set in context of current practice and research in the field; identifying further areas of research. |
| Muktar 2015^7^ | Yes -appropriate to answer research question (patient satisfaction), though focus was broader than PIFU (included satisfaction with clinic FU received). | Partially/no. Patients had no direct experience of PIFU. Unclear how representative patients were of a potentially eligible patient group. Proportion contributing to qualitative data unclear. | Yes/partially. Qualitative data limited to a ‘free text’ comment section as part of a questionnaire. Anonymised data. | N/A Anonymised questionnaire. | Yes. Survey categorised as a service evaluation, therefore formal ethical approval not required. Patients  were provided with an information sheet. | Partially/no. No details on how themes were derived or how data were selected for presentation. Lack of consideration of researcher role in influencing analysis. Potential bias towards routine care in how information was presented to patients on different FU options. | Insufficient detail.  Unclear what proportion of qualitative data presented.  Unclear if more than one analyst involved. | Valuable in terms of: representativeness of findings considered;  findings set in context of current practice and research in the field. |
| Sharma 2020^8^ | Yes/partially -appropriate to answer research question (patient satisfaction with PIFU). Survey with option for free text comments so limited qualitative focus. | Yes/unclear. All women who had received treatment and PIFU during specified time period included in survey but unclear how many provided free text comments. | Yes/partially. Qualitative data limited to ‘free text’ comments as part of a telephone survey. | No details. Surveys conducted by clinical nurse specialists via telephone. | No details. | No details. | No details.  Unclear what proportion of qualitative data presented.  Unclear if more than one | Valuable in terms of:  findings set in context of current practice and research in the field. |
| Williamson 2020^9^ | Yes - to explore views and experiences | Can't tell. Says convenience and snowball sampling used but it is unclear how they chose who to invite so there is potential bias. They did aim for geographical spread across UK but no other purposive sampling mentioned. Not clear if participants were from the same institutions or not. | Likely yes, although there is very little detail of the interview process but the topics seem appropriate. Doesn't say where they were conducted. They acknowledge that data may be out of date (5 years old). | Yes/partially as they did not know each other, but no other consideration of bias from the researcher. | Yes, ethical approval given and consent taken. | Yes/partially - description of analysis is brief with little indication of how codes were derived. Two researchers did analysis who were not those who did the interviews - reason for this is unclear. | Yes, good argument for the results presented, and detailed reporting of results. They don't really discuss the credibility of results (limitations section is very limited). | Clear implications and achieved its aims. |

1. Beaver K, Martin-Hirsch P, Williamson S, et al. Exploring the acceptability and feasibility of patient-initiated follow-up for women treated for stage I endometrial cancer. *Eur J Oncol Nurs* 2020;44:101704. doi: 10.1016/j.ejon.2019.101704 [published Online First: 2019/12/10]

2. Brown L, Payne S, Royle G. Patient initiated follow up of breast cancer. *Psychooncology* 2002;11(4):346-55. doi: 10.1002/pon.576 [published Online First: 2002/08/31]

3. Koinberg IL, Holmberg L, Fridlund B. Breast cancer patients' satisfaction with a spontaneous system of check-up visits to a specialist nurse. *Scand J Caring Sci* 2002;16(3):209-15. doi: 10.1046/j.1471-6712.2002.00040.x [published Online First: 2002/08/23]

4. Kumarakulasingam P, McDermott H, Patel N, et al. Acceptability and utilisation of patient-initiated follow-up for endometrial cancer amongst women from diverse ethnic and social backgrounds: A mixed methods study. *Eur J Cancer Care (Engl)* 2019;28(2):e12997. doi: 10.1111/ecc.12997 [published Online First: 2019/02/13]

5. Lorenc A, Wells M, Fulton-Lieuw T, et al. Clinicians' Views of Patient-initiated Follow-up in Head and Neck Cancer: a Qualitative Study to Inform the PETNECK2 Trial. *Clinical Oncology* 2021 doi: <https://dx.doi.org/10.1016/j.clon.2021.11.010>

6. Moore L, Matheson L, Brett J, et al. Optimising patient-initiated follow-up care - A qualitative analysis of women with breast cancer in the UK. *Eur J Oncol Nurs* 2022;60:102183. doi: 10.1016/j.ejon.2022.102183 [published Online First: 2022/08/07]

7. Muktar S, Thiruchelvam P, Hadjiminas D. Patients' Views of Follow-Up Care After Treatment for Breast Cancer: A Comparison of 2 Approaches. *Journal of Oncology Navigation & Survivorship* 2015;6(6):22-29.

8. Sharma T, Sharma S, Eastwood J, et al. Patient satisfaction with patient-led follow-up for endometrial cancer. *Br J Nurs* 2020;29(17):s4-s10. doi: 10.12968/bjon.2020.29.17.S4 [published Online First: 2020/09/26]

9. Williamson S, Beaver K, Langton S. Exploring health care professionals views on alternative approaches to cancer follow-up and barriers and facilitators to implementation of a recovery package. *Eur J Oncol Nurs* 2020;46:101759. doi: 10.1016/j.ejon.2020.101759 [published Online First: 2020/04/26]
