# Supplementary material for: Systematic review of patients’ and healthcare professionals’ views on patient‐initiated follow‐up in treated cancer patients
Source: Cancer Med. 2023 Jun 16;12(15):16531–47. doi: 10.1002/cam4.6243 (PMC10469665; doi:10.1002/cam4.6243)
Supplement: Supplementary file 3 — Data S3. [file CAM4-12-16531-s007.docx]

**Survey characteristics and findings**

| **Author, year, country, study design** | **Treated cancer** | **Type of PIFU if experienced/**  **questionnaire items** | **Details on questionnaire design (related to PIFU)** | **Sampling, no. of patients, response rate, age/sex, ethnicity, representativeness*** | **Questions and main findings (related to PIFU only)** |
| --- | --- | --- | --- | --- | --- |
| ***Head and neck cancer*** | | | | | |
| Brennan 2019^1^, Canada  *Survey* | Larynx, oropharynx,  hypopharynx and oral cavity | One hypothetical question from wider questionnaire on follow‐up needs of patients and their preferences for organisation of follow‐up care:  *“If there was clear evidence that an earlier diagnosis of a recurrence found at routine follow‐up appointment such as this one did not improve survival compared to a delayed diagnosis at a later appt. or when you had symptoms, what follow‐up would you prefer?”* | Question from Dutch questionnaire developed in a former study (de Bock et al., 2004), modified and translated into English. | Patients completed a questionnaire at routine follow‐up appointments one year after curative treatment; 175 consecutive patients; 88.4% response rate. 83.4% male, mean age 62.5 (10.2). No details on ethnicity. No details on those that declined participation.  Sample may not be representative of urban settings, as recruitment mainly from small towns/villages. | Preferred FU:  No FU appointment: 2 (1.2%)  **Appointments only if I have questions or concerns: 10 (5.7%)**  Less frequent appointments: 23 (13.1 %)  **Continue this (routine) follow‐up program: 140 (80%)**  **Most patients (80%) preferred to continue with their current FU routine** even after explaining that survival is not improved with finding asymptomatic recurrence at an earlier follow‐up appointment compared to symptomatic recurrence at a later appointment. |
| Flanagan 2011^2^, UK  *Conference abstract only*  *Survey* | Oral cancer | Hypothetical question regarding PIFU. All patients had routine FU. | No details. | 64 patients, no details on sampling, response rate or patient characteristics.  Representativeness of sample unclear. | 97% of patients feel they are being reviewed an  appropriate amount and the remaining 3% think they are followed up too frequently.  **83% of patients prefer to be seen regularly rather than only making an appointment if they had any concerns** and 67% would like to be followed up for more than 5 years. 28% of patients had unscheduled appointments, 78% of these were concerns about a lump and 22% were infections. 100% of patients found it easy to make these appointments. |
| Meregaglia 2017^3^, Italy  *Survey/best-worst choice experiment* | Oropharynx, nasopharynx, larynx, oral cavity, sinonasal cavity, salivary glands or other H&N cancer | Patients in routine FU asked about hypothetical scenarios.  Patients asked about frequency and length of FU; frequency of MRI/CT scans; frequency and eligibility of PET scans; and telephone calls to monitor occurrence of new symptoms (including no intervisit calls). | Scenarios developed based on literature review to identify studies that assessed patients’ preferences around post-treatment programs in oncology using stated preference methods. Interviews with six patients during routine hospital visits used to refine terminology and evaluate  comprehension and acceptability. | Consecutive sample of patients during routine FU appointment; 143/162 (90%) response rate. 74% male, mean age 57.6 (12.1). No details on ethnicity.  Authors suggest that due to nature of cancer centre, recruited from, patients may be more educated, wealthy or health conscious than a general H&N cancer population. | Not assessing PIFU specifically, but relates to symptom driven scans:  Most preferred scenario: hospital-based FU with frequency of visits decreasing over time, radiological assessments (MRI/CT) once or twice a year, yearly PET irrespective of individual risk of recurrences), and intervisit calls by the oncologist to monitor the occurrence of new symptoms.  **Least preferred scenario is mixed hospital-based/primary care–based surveillance, with MRI/CT scan only at the occurrence of new symptoms**, no PET scan scheduled during the FU period, and intervisit calls by the nurse. |
| Mueller 2019^4^, Switzerland  *Survey/hypothetical scenarios* | Head and neck squamous cell carcinoma  Most with tumour or oral cavity, larynx or oropharynx. | Detailed hypothetical FU scenarios, with more/less scheduled imaging/imaging only in case of symptoms.  Q1  *“Would you prefer to make appointments only when you experience new symptoms, instead of scheduled exams?”*  Q2  *“Would you accept to participate in a study in which you would be randomly assigned to one of the presented follow-up schedules”?* | Scenarios and questions created by authors; no further details. | Consecutive sample during scheduled clinic visits; 101/110 (92%). 70.3% male, mean age 64.3 (9.1). No details on ethnicity.  Sample from single tertiary cancer centre may not be representative of wider H&N cancer population. | Type of FU scenario preferred:  Over half preferred fewer FU visits (provided there is regular imaging).  85% of patient preferred FU with scheduled imaging.  Q1  **89.1% preferred structured FU with scheduled examinations.**  **7.8% favoured self-referral.**  Q2  66% would participate in a study with random assignment to a FU schedule. (Increased to 97% if could choose FU schedule)  23.8% would not participate.  9.9% were unsure.  NB patients not questioned directly at start pf FU period |
| Trinidade 2012^5^, UK  *Survey* | Head and neck cancer | Views on routine FU experienced, and hypothetical questions on less intensive FU system based on patients reporting problems and requesting appointments. | No details. | 263 patients  consecutively attending a head and neck cancer clinic. No details on response rate or patient characteristics.  Representativeness of sample unclear. | 84% felt that FU visits were too frequent.  84% of those also booked to see an allied health professional (on same day as clinic) felt that issues addressed were duplicated/overlapped.  **73% were on favour of a less intensive FU system based on patients reporting problems and requesting appointments.** The preferred contact in such a system was (in order of preference) clinical nurse specialist, medical secretary, clinician, speech & language therapist, GP, dietician. |
| ***Breast cancer*** | | | | | |
| Al-Gailani 2010^6^, UK  *Conference abstract only*  *Survey* | Breast cancer | Survey on preferences for type of FU in patients in standard FU. | No details. | Questionnaire provided to 75 women attending standard FU at breast cancer clinic.  No details on response rate or patient characteristics.  Representativeness of sample unclear. | Most patients satisfied with current hospital FU.  **56% were willing to be discharged from hospital FU after 3 years providing an open access system is in place.** |
| Brown 2000^7^, UK  *Conference abstract only*  *Structured interview, frequencies analysed* | Breast cancer | Hypothetical questions related to FU, including: *“How likely are women to accept non-clinic, patient-initiated FU with open access to a Breast Care Nurse?”* | No details. | 120 women approached for interview (of 470 attending regular breast clinics for FU appointments); response rate 100/120 (83%)  Mean age 64 (43-93), no details on ethnicity.  Representativeness of sample unclear. | **90% of women wished to continue clinical FU, 8% would accept PIFU FU**, 2% preferred a combination of the two.  7% would accept PIFU immediately, 34% claimed they would never accept it. |
| Chapman 2009^8^, UK  *Single arm cohort, survey* | Breast cancer with low risk of recurrence | Patients selected for PIFU by multidisciplinary team. Symptoms and signs of recurrence discussed, and contact details for specialist nurses provided to allow rapid self-referral to the breast clinic if concerns arose. Regular mammograms scheduled.  Questionnaire items on whether information received/ ease of understanding of information, on accessing breast unit and overall satisfaction. | No details. | Questionnaire sent to 217 women who had taken part in PIFU. Response rate: 130/217 (60%) patients completed at least one of 10 questions.  No details on age or ethnicity.  Representativeness of sample unclear. | **106/106 (100%) very satisfied or satisfied with PIFU service.** (NB 18% did not answer this question)  13/130 (10%) thought PIFU service could be improved  The vast majority understood the information provided (verbally or written) and had a clear idea of how to contact the breast unit. |
| Gulliford 1997^9^, UK  *RCT, including survey of FU preferences (preliminary data)* | Any stage of breast cancer | Not denoted as PIFU, but patients received mammograms only (compared to routine scheduled visits + mammogram). All patients could request visits based on symptoms. | No details on how questions derived. | Women taking part in RCT (n=196). Age between <49 to >65 (around half 50-65); no details on ethnicity.  Those declining RCT participation (7%) had a higher stage of primary disease, were younger and with a more recent diagnosis. | 94% (routine FU) vs 88% (mammogram only) found clinic visits reassuring.  94% (routine FU) vs 89% (mammogram only) wished to continue with hospital FU rather than GP FU alone.  **11% (routine FU) vs 16% (mammogram only) preferred a more frequent visit schedule.**  **25% (routine FU) vs 35% (mammogram only) preferred a less frequent visit schedule.** |
| Koinberg 2004^10^, Sweden  *RCT, including survey of patient satisfaction* | Stage I or II breast cancer | Patients taught by nurse how to recognise symptoms of recurrence, and advised to contact nurse in event of symptoms or other questions. Mammography at 1-year intervals for 3 years.  Questionnaire on patient satisfaction with the follow-up system and accessibility of breast medical services including whether or  not the patient wanted to change the follow-up  routines. | Questionnaire specifically designed/adapted for this study; previously used in similar study in men with prostate cancer. Six questionnaires over 3-year period. | Women taking part in RCT of on-demand nurse-led FU compared with routine FU. 400 patients randomised, but only 66% in final analysis (one centre with n=135 excluded as services deemed to be too similar). Mean age 60 and 58.8, no details on ethnicity.  Representativeness of sample unclear. | **No statistically significant differences between groups in terms of patient satisfaction** (between 93-100% depending on time-point and group).  No statistically significant differences between groups in terms of experiences of accessibility. |
| Muktar 2015^11^, UK  *Survey including a “free text” comments section (see qualitative data)* | Breast cancer (any stage) | Questionnaire about future preferences for FU, including “open -access FU” in patients undergoing standard FU. | Questionnaire designed and piloted in the breast surgery department; categorised as service evaluation (no ethical approval required). No further details. | All eligible women at one hospital who had received at least 6 months of standard FU were invited to complete a questionnaire during a 6-month period. 317 women recruited (number invited not stated). Age not stated. 78% Caucasian, 9% Afro-Caribbean, 7% Indo-Asian.  Authors suggest that patients are broadly representative, though self-selection bias may be an issue. | **91% (264/290) favoured standard**  **clinical FU compared with open-access FU**  63% (173/273) noted that open-access FU is ineffective.  92% (269/292) reported that existing clinical-led FU was effective.  89% (271/304) requested that their FU be led by a breast surgeon and oncologist rather than their primary care physician or a community nurse.  No significant correlation observed between  the type of FU preferred and age, ethnic background,  distance from hospital, or time since diagnosis. |
| Riis 2020^12^  Denmark  *RCT including questionnaire* | Hormone-receptor  Positive, early stage breast cancer (stage I-III) | No routine consultations (apart from administration of medications).  Patient could request additional consultation or phone call with a specialized nurse (via e-questionnaire, email, or telephone call). | Four items from Patient Experience Questionnaire (PEQ) | All women enrolled in RCT (recruited from one oncology department), n=136 (65 in PIFU group); 124 analysed (64 in PIFU group)  Mean age 64.4; no details on ethnicity.  35% eligible women excluded due to lack of technical skills/access to computer, co-psychosocial comorbidities, cognitive impairment or personal reasons. | In PIFU group:  Proportion satisfied or very satisfied with FU:>88% at each time point  Proportion that wished for procedures that were not offered: 1-7% (depending on time-point; estimated from graph)  Proportion that had a concern that did not lead to contact with the department: 5-17% (depending on time-point; estimated from graph)  Proportion that would have liked more information on something: 4-18% (depending on time-point; estimated from graph)  NB similar proportions to control group. |
| Walder 2020^13^, UK  *Conference abstract only*  *Survey* | Low risk breast cancer | Open-access patient-led follow-up. Patients monitor own symptoms and contact clinic if concerned. | No details | Pre- and post-appointment questionnaires distributed to patients attending a nurse-led post-treatment holistic clinic. 69 responses (no details on response rate).  No further details on participants. | Pre-appointment: 71.6% of patients felt they already understood the open-access system.  Post-appointment: 98.4% understood the reasoning of the open access system and were happy  9.5% were not confident identifying symptoms which  might warrant follow-up (group spanned all age groups and treatment modalities). |
| Whitehead 2019^14^, UK  *Conference abstract only*  *Survey* | Breast cancer | Patients in standard FU and supported self-management (SSM) FU asked about understanding of surveillance protocols and signs of recurrence, contact and whether concerns were addressed. | No details. | 223 women from supported SSM programme (64% response rate) and 60 from standard FU (97% response rate). Age or ethnicity not stated.  No further details on how patients selected.  Representativeness of sample unclear. | **SSM comparable to standard FU** in terms of understanding the surveillance protocols (98% SSM vs 97% clinic FU), addressing concerns (94% vs 100%), contact (98% vs 97%) and understanding signs of recurrence (98% vs 93%). |
| ***Endometrial cancer*** | | | | | |
| Beaver 2020^15^, UK  *Survey* | Stage I endometrial | Patients had taken part in a trial on hospital versus telephone follow-up, so had no experience of PIFU.  As part of the wider survey patients were asked to rank 8 FU scenarios. One of these was:  *“I would prefer to be discharged from follow-up after treatment as long as I know who to contact if I have a problem.”* | Questions derived from previous questions used in a study on nurse-led telephone follow-up for women with breast cancer (Beaver et al. 2009) and the ENDCAT trial (Beaver et al. 2017). | 211/236 (89.4%) women from the ENDCAT trial. Mean age 64.8 (8.8), 99% White.  Not representative of ethnic diversity in UK. | Patients who had been in hospital FU:  The most highly ranking types of FU were hospital appointments with a doctor and/or specialist nurse (ranks 1,2,3). **The following scenario was ranked 4^th^: *“I would prefer to be discharged from follow-up after treatment as long as I know who to contact if I have a problem.”***  Patient who had been in telephone FU:  The most highly ranking types of FU were hospital appointments with a doctor and/or telephone appointments with a specialist nurse, followed by hospital appointments with a nurse and/or doctor. The open access option was ranked 6^th^. |
| Beaver 2020^16^, UK  *Mixed methods study (see also qualitative studies)* | Stage I endometrial | Patients took part in PIFU, supported by a self-management approach. Information given on signs and symptoms of recurrence and who to contact. Hospital-based appointment at the end of the study.  Aims was to assess feasibility and acceptability of PIFU.  Patients asked about 12 FU options and rated these on a Likert scale. | No details. | Gynaecology oncologists and clinical nurse specialists identified 65 eligible suitable women from their clinics, 17 (26%) agreed to participate (mean age 59.41 (SD 10.82, range 34–79), 94% White.  12/17 (71%) responses at FU.  Participants mainly white, well educated, from higher socio-economic groups and younger than average. | Findings at recruitment (T1) and after (minimum) 6 months FU (T2).  Most popular options at T1 were a **mix of hospital and telephone appointments (59% indicated ‘like a lot/like’),** **early discharge from hospital supported by the Recovery Package (59%)**, FU appointments with a GP (59%), and telephone appointments with a specialist nurse (59%).  41-47% expressed support for hospital appointments (with different health professionals).  At T2, the **most popular options were early discharge from hospital supported by the Recovery Package** (58%) and FU appointments with a GP (58%).  33-50% expressed support for hospital appointments (with different health professionals).  At T1 and T2, **18% and 25% respectively indicated that they disliked early discharge from hospital with the Recovery Package.**  12/17 (71%) said they would be willing to be randomised in a future trial of PIFU at T1, and 9/12 (75%) at T2. |
| Kumarakulasingham 2019^17^, UK  *Mixed methods study (see also qualitative studies* | Early stage endometrial | Patients took part in PIFU. Contact details of clinical nurse specialist provided at end of treatment appointment, as well as written information on signs and symptoms that should prompt medical review. Telephone calls at 6 and 12 months to ensure patient was happy to continue on PIFU and had contact details.  Patient satisfaction questionnaire (on contact, advice given and symptoms) included:  *“Overall how reassured are you by PIFU?”* | No details. | 228 randomly sampled women enrolled on PIFU scheme (either transferred from routine hospital follow‐up or directly into PIFU). Median age 65 (42–90). 89.5% White, 9.6% South Asian, 0.9% African/AfroCaribbean. Response rates for questionnaires at 6 and 12 months unclear.  Representativeness of sample unclear, though likely to include a proportion of non-White women. | PIFU was mostly well received. 62% found PIFU useful (very much/quite a bit), 0.6% (1 patient) not useful at all (at 6 months).  Four women (3.6%) found the scheme not reassuring at all at 12 months, but **63% of women were reassured (very much/quite a bit) by PIFU at 12 months**. |
| Sharma 2020^18^, UK  *Survey* | Surgically treated endometrial cancer | Patient took part in patient-led telephone follow-up, where they could call the clinical nurse specialist team at any time if they had any concerns. | No details.  NB Satisfaction question based on entire treatment rather than on the telephone follow-up service specifically. | 104 women (83 patients  white British, 3 Asian, 2 mixed ethic origin, 16 did not disclose ethnicity).  Age range 34-89 years (most aged 61-80 years)  94 (90%) responded to survey. | 97% of patients knew to contact a health professional with any concern regarding their cancer.  78% would contact the clinical nurse specialist (CNS) about concerns.  19% would contact their GP about concerns.  91% had a contact number for the clinical nurse specialist.  90% remembered symptoms suspicious for recurrence.  **92 % of patients scored 9/10 or 10/10 on the satisfaction survey (*NB this was based on entire treatment).*** |
| **Breast or gynaecological cancer** | | | | | |
| Pakiz 2019^19^, Slovenia  *Survey* | Breast or gynaecological cancers | Questionnaire items on hypothetical future follow-up:  Whether regular visits make patients feel safe and/or stressed.  Whether patients would prefer to have no regular visits and visit only when symptoms occur. | No details. | Consecutive women who completed primary treatment of gynaecological or breast cancer, attending every fourth follow-up  outpatient office during a three-month period. 122/150 (81%) invited patients and 72/75 invited physicians. Mean patient age 60.3 ± 12.3. No details on ethnicity. Physicians were GPs or primary care gynaecologists.  Representativeness of sample unclear. | Patients:  92% thought that regular visits made them safer.  36% described regular visits as a source of stress.  **22% expressed highest level of agreement regarding symptom led appointments** (highest in 51-69 year olds, lowest in >70 years olds).  Physicians:  65% thought that patients would feel safer with regular visits.  14% thought that regular visits would cause patients stress.  Only **1.4% thought that patients would like to make their own appointments.** |
| **Colorectal cancer** | | | | | |
| Batehup 2017^20^, UK  *Prospective cohort study* | Colorectal | Patients self-management workshop at treatment completion; no further routine FU appointments; surveillance blood tests 3 monthly for 2 years; 6 monthly years 3-5. Computed Tomography at 1,2,5 years; colonoscopy at 1, 5 years unless abnormal.  Standard QoL questionnaires, also tool on FU care and patient ratings (on whether care was acceptable; met expectations; perception of service quality) | Tool on FU care developed for study, from a review of FU evaluations (scale 0-100); no details on patient rating questions. | Patients in study comparing PIFU with traditional outpatient FU (n=363). Mean age around 72; 36-43% female. Between 97-100% White. Response rates between 45 and 74% (depending on group and time-point- FU over 1 year).  Representativeness of sample unclear. | **36/37 (97.3%) of patients in PIFU found their FU to be acceptable compared with 24/32 (75%) in routine FU.**  No significant differences between groups regarding reassurance, quality of care or the extent to which it met expectations; most reported high satisfaction.  NB Patients in routine FU were sicker. |
| Wilkinson 2009^21^, UK | Colorectal | Yearly CT scans for 3 years, colonoscopic surveillance 3-5 yearly. Dedicated hotline number for patients to call should they develop symptoms. Open access clinic via hotline. Information booklet with symptoms provided.  Questions relevant to FU:  *How happy are you with the service the colorectal nurse specialists provide* (NB this includes time from diagnosis onwards)?  *Do you feel it is helpful to have a telephone number to that you ring at any time if you are worried?*  *If you needed to ring this number did you get a prompt and satisfactory response?*  *Are you happy with the present system of appointments?* | Developed for service audit with clinical audit team. Used in previous study in 2004. | 195 patients randomly selected who had undergone potentially curative or palliative treatment. Response rate 65%. No non-English speaking patients or patient from ‘multicultural’ backgrounds. No details on age/sex.  Sample not representative in terms of ethnicity, non-English speaking population. | **88/120 (73%) of patients were happy not to have regular clinic appointments; 32/120 (27%) would have preferred regular clinic visits.**  75% were very happy and 21% fairly happy with the specialist nurse service (NB this includes all aspects of care, not just FU).  120/121 (99%) felt it was helpful to have a telephone number to ring.  63/64 (98%) that needed to ring the number got a prompt and satisfctory reponse. |
| **Any cancer** | | | | | |
| Frew 2010^22^, UK  *Survey* | Not stated (15 medical and surgical specialities) | Patients asked about experiences of FU care and which they would personally prefer.  Primary care practitioners and specialist clinicians asked which model of FU care they would advocate. | Based on literature review and consultation with National Clinical Leads for cancer, the 34 cancer networks  and the Cancer Patient Partnership Forum. Research questionnaires then developed and piloted by clinicians and managers in the Cancer Services  Collaborative Improvement Partnership, with advice from  Picker Europe and 25 service users. | Questionnaires distributed through the 34 cancer networks and all primary care trusts in England. 2928 respondents (604 patients, 22 carers, 883 GPs, 57 practice nurses, 804 doctors and 558 specialist nurses/allied health professionals).  67% (service users) were women. 63% diagnosed and treated within last 5 years. Sample thought to be reasonably representative for age and ethnic background. | % preference for FU in patients/doctors who had experience of that type of FU. Overall sample size: patients (n=626), primary care practitioners (n=940), specialist clinicians (1360). Sample size for those who had experience of PIFU: patients (n=173), primary care practitioners (n=252), specialist clinicians (600).  Outpatient: 86% , 83%, 81%  **Patient managed: 82%, 83%, 86%**  Telephone: 73%, 78%, 82%  Non-specialist: 68%, 79%, 87%  Group: 56%, 58%, 76%  Postal: 32%, 49%, 48%  No follow-up: 7%, 45%, 65%  Other: 63%, 52%, 72%  No data for preferences in those who had no experience of that type of FU. Authors state that *“large differences in scores suggest that the ‘without experience’ results have been affected by a lack of awareness and understanding of the various methods proposed, as opposed to a lack of preference for those methods.”* |

FU=follow-up; *in relation to a wider/general population with the same cancer

1. Brennan KE, Hall SF, Yoo J, et al. Routine follow-up care after curative treatment of head and neck cancer: A survey of patients' needs and preferences for healthcare services. *Eur J Cancer Care (Engl)* 2019;28(2):e12993. doi: 10.1111/ecc.12993 [published Online First: 2019/01/19]

2. Flanagan J, Goodson M, Banks R, et al. Oral cancer patients experience of follow up care. *British Journal of Oral and Maxillofacial Surgery* 2011;1):S56. doi: <http://dx.doi.org/10.1016/j.bjoms.2011.03.093>

3. Meregaglia M, Cairns J, Alfieri S, et al. Eliciting Preferences for Clinical Follow-Up in Patients with Head and Neck Cancer Using Best-Worst Scaling. *Value Health* 2017;20(6):799-808. doi: 10.1016/j.jval.2017.01.012 [published Online First: 2017/06/05]

4. Mueller SA, Riggauer J, Elicin O, et al. Patients' preferences concerning follow-up after curative head and neck cancer treatment: A cross-sectional pilot study. *Head Neck* 2019;41(7):2174-81. doi: 10.1002/hed.25686 [published Online First: 2019/02/07]

5. Trinidade A, Kothari P, Andreou Z, et al. Follow-up in head and neck cancer: patients' perspective. *Int J Health Care Qual Assur* 2012;25(2):145-9. doi: 10.1108/09526861211198308 [published Online First: 2012/03/30]

6. Al-Gailani M, Dudani P, Fletcher M, et al. Breast cancer follow-up: What about the patients' views? *European Journal of Surgical Oncology* 2010;36(11):1119. doi: <http://dx.doi.org/10.1016/j.ejso.2010.08.005>

7. Brown L, Payne S, Royle G. Outpatient follow-up for breast cancer patients. *Psycho-Oncology* 2000;9(363)

8. Chapman D, Cox E, Britton PD, et al. Patient-led breast cancer follow up. *Breast* 2009;18(2):100-2. doi: 10.1016/j.breast.2009.01.006 [published Online First: 2009/02/24]

9. Gulliford T, Opomu M, Wilson E, et al. Popularity of less frequent follow up for breast cancer in randomised study: initial findings from the hotline study. *BMJ* 1997;314(7075):174-7. doi: 10.1136/bmj.314.7075.174 [published Online First: 1997/01/18]

10. Koinberg IL, Fridlund B, Engholm GB, et al. Nurse-led follow-up on demand or by a physician after breast cancer surgery: a randomised study. *Eur J Oncol Nurs* 2004;8(2):109-17; discussion 18-20. doi: 10.1016/j.ejon.2003.12.005 [published Online First: 2004/06/03]

11. Muktar S, Thiruchelvam P, Hadjiminas D. Patients' Views of Follow-Up Care After Treatment for Breast Cancer: A Comparison of 2 Approaches. *Journal of Oncology Navigation & Survivorship* 2015;6(6):22-29.

12. Riis CL, Jensen PT, Bechmann T, et al. Satisfaction with care and adherence to treatment when using patient reported outcomes to individualize follow-up care for women with early breast cancer - a pilot randomized controlled trial. *Acta Oncol* 2020;59(4):444-52. doi: 10.1080/0284186X.2020.1717604 [published Online First: 2020/02/01]

13. Walder E, Cox E. Evaluating patient attitudes to patient-led follow-up following breast cancer. *European Journal of Surgical Oncology* 2020;46(6):e51. doi: <http://dx.doi.org/10.1016/j.ejso.2020.03.192>

14. Whitehead I, Alfred J, Chagla L. P133. Supporting Survivors to Manage their Cancer Surveillance: Not Merely a Reallocation of Resources. *European Journal of Surgical Oncology* 2019;45(5):919. doi: <http://dx.doi.org/10.1016/j.ejso.2019.01.153>

15. Beaver K, Williamson S, Sutton CJ, et al. Endometrial cancer patients' preferences for follow-up after treatment: A cross-sectional survey. *Eur J Oncol Nurs* 2020;45:101722. doi: 10.1016/j.ejon.2020.101722 [published Online First: 2020/02/06]

16. Beaver K, Martin-Hirsch P, Williamson S, et al. Exploring the acceptability and feasibility of patient-initiated follow-up for women treated for stage I endometrial cancer. *Eur J Oncol Nurs* 2020;44:101704. doi: 10.1016/j.ejon.2019.101704 [published Online First: 2019/12/10]

17. Kumarakulasingam P, McDermott H, Patel N, et al. Acceptability and utilisation of patient-initiated follow-up for endometrial cancer amongst women from diverse ethnic and social backgrounds: A mixed methods study. *Eur J Cancer Care (Engl)* 2019;28(2):e12997. doi: 10.1111/ecc.12997 [published Online First: 2019/02/13]

18. Sharma T, Sharma S, Eastwood J, et al. Patient satisfaction with patient-led follow-up for endometrial cancer. *Br J Nurs* 2020;29(17):s4-s10. doi: 10.12968/bjon.2020.29.17.S4 [published Online First: 2020/09/26]

19. Pakiz M, Lukman L, Kozar N. Patients’ and physicians’ expectations differ significantly during the follow-up period after completion of primary treatment of gynecological or breast cancer. *Eur J Gynaecol Oncol* 2019;5

20. Batehup L, Porter K, Gage H, et al. Follow-up after curative treatment for colorectal cancer: longitudinal evaluation of patient initiated follow-up in the first 12 months. *Support Care Cancer* 2017;25(7):2063-73. doi: 10.1007/s00520-017-3595-x [published Online First: 2017/02/16]

21. Wilkinson S, Sloan K. Patient satisfaction with colorectal cancer follow-up system: an audit. *Br J Nurs* 2009;18(1):40-4. doi: 10.12968/bjon.2009.18.1.32089 [published Online First: 2009/01/08]

22. Frew G, Smith A, Zutshi B, et al. Results of a quantitative survey to explore both perceptions of the purposes of follow-up and preferences for methods of follow-up delivery among service users, primary care practitioners and specialist clinicians after cancer treatment. *Clin Oncol (R Coll Radiol)* 2010;22(10):874-84. doi: 10.1016/j.clon.2010.06.008 [published Online First: 2010/07/10]
