# Supplementary material for: Systematic review of patients’ and healthcare professionals’ views on patient‐initiated follow‐up in treated cancer patients
Source: Cancer Med. 2023 Jun 16;12(15):16531–47. doi: 10.1002/cam4.6243 (PMC10469665; doi:10.1002/cam4.6243)
Supplement: Supplementary file 4 — Data S4. [file CAM4-12-16531-s003.docx]

**Qualitative studies: conference abstracts**

| **Author, year, country** | **Focus of study, type of cancer** | **Participants and recruitment** | **Data collection method, setting, analysis method** | **Number of participants, m/f, age** | **Time since treatment end** | **Description of PIFU** | **Major themes related to PIFU** | **Comments** |
| --- | --- | --- | --- | --- | --- | --- | --- | --- |
| Amirthanayagam 2021a^1^, UK | To gain an understanding of clinicians’ views on **endometrial cancer**  follow-up strategies. | Gynaecological  oncologists, cancer unit gynaecologists, oncologists and clinical nurse specialists.  Purposeful sampling to ensure diverse clinical backgrounds, experience and UK geography. | Semi-structured telephone interviews. Framework analysis. | No details. | Not applicable to participants. | No details. | Overwhelming interest in PIFU schemes and development of a national follow-up strategy.  Variation in the structure and patient populations  clinicians felt should be included in the schemes.  Addition of biomarker monitoring would increase the confidence in  transferring patients with high-risk or advanced disease. | None. |
| Amirthanayagam 2021b^2^, UK | To evaluate the impact of covid-19 on **endometrial cancer**  follow-up strategies. | Gynaecological  oncologists, cancer unit gynaecologist, oncologists and clinical nurse specialists involved in follow-up of patients after diagnosis of endometrial  cancer. | Survey and qualitative interview study.  Interviews conducted virtually and analysed using. framework  analysis. | No details. | Not applicable to participants. | No details. | Support to reduce face to-  face consultations during the COVID-19 pandemic and strong support for telephone and patient- initiated follow-up. | None. |
| Calman 2011^3^  UK | Gain an understanding of health professionals' views on follow-up for **lung cancer** and integrate these into a new intervention for follow-up | Health professionals.  Purposeful sampling from range of professional backgrounds (clinical & medical oncologists, surgeons, palliative care doctors, nurse specialists) | 17 interviews, setting not stated.  Grounded theory; constant comparative method. | Not stated | Not applicable to study participants. Unclear for patients. Focus on FU after treatment, but appears to include patients with advanced cancer and palliative care. | No specific PIFU programme-general views on FU. | *"Interviews highlighted* ***clinicians’ preference*** *for regular HCP initiated appointments; participants expressed* ***concern that a patient initiated system would lead vulnerable patients to ‘fall through the net’ and miss opportunities for supportive care or ‘miss the boat’ for further treatment****."* | The authors note that the disease trajectory for lung cancer is often short with rapid deterioration, so patient led, less intensive models of care were felt to be unsuitable. |
| Drabarek 2021^4^, Australia | Gain an understanding of patient experiences of **melanoma** self-surveillance using mobile digital technology | Patients.  Qualitative study nested within a pilot randomised controlled trial (no further details) | Semi-structured interviews and theoretically driven thematic analysis (using extended technology acceptance model framework). Melanoma centres in New South Wales. | 20 patients (8 female/ 12 male); median age 62 | No details | Patient-led surveillance using a mobile dermatoscope and accompanying app for sending images (in addition to usual care). | Facilitators: increased access to care; enhancement of early detection; increased awareness of skin and importance of skin self-examination.  Barriers: learning to use the tools with confidence requires practice; patients and skin check partner require educational and technical support, particularly if they have many moles or low technology self-efficacy; lack of a skin check partner. | None. |
| Fairley 2020^5^, UK | To determine  the views and opinions on the acceptability of PIFU in **gynaecological cancer** patients. | Gynaecological cancer patients and carers. | Semi-structured interviews | 58 participants (no further details). | No details. | No details (‘patient initiated follow up’). | Facilitators:  None mentioned.  Barriers:  Patients thought hospital appointments were for detecting recurrence and found them reassuring; did not want telephone or skype FU. | Patients had no experience of PIFU. |
| Timmons 2015^6^  Ireland/  UK | Gain an understanding of health professionals' views on alternative models of follow-up in **prostate cancer**, including PIFU | Multi-disciplinary health professionals involved in prostate cancer follow-up. | In-depth, semi-structured interviews, setting not stated.  Extended Normalisation Process Theory framework | 23 HCPs, no further details | Not applicable to study participants. 'Post-treatment' for patients. | No specific PIFU programme-general views on FU. | All models of FU discussed were deemed acceptable to an extent. Nurse-led follow-up was associated with fewer perceived barriers. | Views on PIFU specifically not reported. |

1. Amirthanayagam A, Jones E, Moss E. Clinicians' views on endometrial cancer follow-up strategies. *International Journal of Gynecological Cancer* 2021;31(SUPPL 1):A181. doi: <https://dx.doi.org/10.1136/ijgc-2021-ESGO.309>

2. Amirthanayagam A, Boulter L, Morris H, et al. Impact of COVID 19 on endometrial cancer follow-up strategies. *International Journal of Gynecological Cancer* 2021;31(SUPPL 1):A190. doi: <https://dx.doi.org/10.1136/ijgc-2021-ESGO.326>

3. Calman L, Beaver K, Roberts C. Health care professionals' views of the follow-up of lung cancer patients. *European Journal of Cancer* 2011;1):S322-S23.

4. Drabarek D, Habgood E, Janda M, et al. Patient experiences of melanoma self-surveillance using mobile digital technology. *Asia-Pacific Journal of Clinical Oncology* 2021;17(SUPPL 5):22. doi: <http://dx.doi.org/10.1111/ajco.13651>

5. Fairley H, Anderson Y, Ang C, et al. Patient initiated follow up: Experiences from a tertiary gynaecological oncology centre. *International Journal of Gynecological Cancer* 2020;30(SUPPL 3):A160. doi: <https://dx.doi.org/10.1136/ijgc-2020-IGCS.331>

6. Timmons A, Gooberman-Hill R, Gallagher P, et al. Health professional's views about the acceptability and implementation of alternative models of prostate cancer follow-up. *BJU International* 2015;2):34. doi: <http://dx.doi.org/10.1111/bju.13221>
