# Supplementary material for: Systematic review of patients’ and healthcare professionals’ views on patient‐initiated follow‐up in treated cancer patients
Source: Cancer Med. 2023 Jun 16;12(15):16531–47. doi: 10.1002/cam4.6243 (PMC10469665; doi:10.1002/cam4.6243)
Supplement: Supplementary file 5 — Data S5. [file CAM4-12-16531-s001.docx]

**Sample search strategy in Embase**

1 (patient$ adj2 initiat$).ti,ab.

2 (patient$ adj2 prefer$).ti,ab.

3 (patient$ adj2 request$).ti,ab.

4 (patient$ adj2 prompt$).ti,ab.

5 (patient$ adj2 trigger$).ti,ab.

6 (patient$ adj2 led).ti,ab.

7 (patient$ adj2 driv$).ti,ab.

8 (patient$ adj2 generat$).ti,ab.

9 (survivor$ adj2 initiat$).ti,ab.

10 (survivor$ adj2 prefer$).ti,ab.

11 (survivor$ adj2 request$).ti,ab.

12 (survivor$ adj2 prompt$).ti,ab.

13 (survivor$ adj2 trigger$).ti,ab.

14 (survivor$ adj2 led).ti,ab.

15 (survivor$ adj2 driv$).ti,ab.

16 (survivor$ adj2 generat$).ti,ab.

17 self-refer$.ti,ab.

18 help seek$.ti,ab.

19 helpseek$.ti,ab.

20 follow up$.ti,ab.

21 clinic$.ti,ab.

22 consultation$.ti,ab.

23 appointment$.ti,ab.

24 monitor$.ti,ab.

25 aftercare.ti,ab.

26 refer$.ti,ab.

27 examination$.ti,ab.

28 surveillance.ti,ab.

29 watchful waiting.ti,ab.

30 survivorship care.ti,ab.

31 survivorship plan$.ti,ab.

32 (follow-up adj2 strateg$).ti,ab.

33 (follow-up adj2 model$).ti,ab.

34 (follow-up adj2 system$).ti,ab.

35 (follow-up adj2 protocol$).ti,ab.

36 (follow-up adj2 pathway$).ti,ab.

37 (individuali#ed adj2 follow-up).ti,ab.

38 (targeted adj2 follow-up).ti,ab.

39 personali#ed follow-up.ti,ab.

40 (follow-up adj2 prefer$).ti,ab.

41 personali#ed care.ti,ab.

42 individuali#ed care.ti,ab.

43 personali#ed survivorship.ti,ab.

44 individuali#ed survivorship.ti,ab.

45 (prefer$ adj2 survivorship).ti,ab.

46 cancer$.ti,ab.

47 neoplasm$.ti,ab.

48 carcinoma$.ti,ab.

49 metastas$.ti,ab.

50 malignan$.ti,ab.

51 tumo?r$.ti,ab.

52 exp malignant neoplasm/

53 (patient$ adj3 experience$).ti,ab.

54 interview$.ti,ab.

55 (patient$ adj3 perspective$).ti,ab.

56 (patient$ adj3 opinion$).ti,ab.

57 survey$.ti,ab.

58 questionnaire$.ti,ab.

59 acceptabilit$.ti,ab.

60 (patient$ adj3 satisfaction$).ti,ab.

61 qualitative$.ti,ab.

62 mixed method$.ti,ab.

63 exp qualitative research/

64 exp grounded theory/

65 exp thematic analysis/

66 exp ethnography/

67 exp patient attitude/

68 grounded theor$.ti,ab.

69 thematic analys$.ti,ab.

70 ethnograph$.ti,ab.

71 (patient$ adj3 attitude$).ti,ab.

72 (patient$ adj3 belie$).ti,ab.

73 (patient$ adj3 behavio$).ti,ab.

74 exp health behavior/

75 exp health belief/

76 focus group$.ti,ab.

77 1 or 2 or 3 or 4 or 5 or 6 or 7 or 8 or 9 or 10 or 11 or 12 or 13 or 14 or 15 or 16 or 17 or 18 or 19

78 20 or 21 or 22 or 23 or 24 or 25 or 26 or 27 or 28 or 29 or 30 or 31

79 77 and 78

80 32 or 33 or 34 or 35 or 36 or 37 or 38 or 39 or 40 or 41 or 42 or 43 or 44 or 45

81 79 or 80

82 46 or 47 or 48 or 49 or 50 or 51 or 52

83 81 and 82

84 53 or 54 or 55 or 56 or 57 or 58 or 59 or 60 or 61 or 62 or 63 or 64 or 65 or 66 or 67 or 68 or 69 or 70 or 71 or 72 or 73 or 74 or 75 or 76

85 83 and 84
