# Supplementary material for: Systematic review of patients’ and healthcare professionals’ views on patient‐initiated follow‐up in treated cancer patients
Source: Cancer Med. 2023 Jun 16;12(15):16531–47. doi: 10.1002/cam4.6243 (PMC10469665; doi:10.1002/cam4.6243)
Supplement: Supplementary file 6 — Data S6. [file CAM4-12-16531-s006.docx]

**Excluded studies with reasons**

| **Study** | **Full text or conference abstract** | **Reason for exclusion** |
| --- | --- | --- |
| Alders & Hermens 2017. Towards personalized surveillance for head and neck cancer patients. Journal of Clinical Oncology, Volume 35, Issue 31_suppl 116 | Conference abstract | Follow-up preferences of patients explored, but does not include any reference to PIFU. |
| Bourne et al. Defining the Components and Piloting of a Self- Management Workshop for Testicular Cancer Survivors: They’ll think I’m not Going to a Workshop But They’ll Benefit From It  &  Bourne et al. Defining and Piloting a Self-Management Workshop for Colorectal Cancer Survivors. Psycho-Oncology 2021; 21(Suppl. 2) | Conference abstracts | Evaluation and piloting of self-management workshop. No data of PIFU specifically. |
| Coleman L, Newton C. Patient initiated follow up after gynaecological malignancy: National survey of current UK practice. Eur J Obstet Gynecol Reprod Biol. 2020 May;248:193-197. doi: 10.1016/j.ejogrb.2020.03.028. Epub 2020 Mar 19. PMID: 32240892. | Full text | Prevalence/timing of PIFU for different types of cancer. No data (qualitative or quantitative) on patient or health professional views of PIFU. |
| Dahl et al. Paradoxes of follow-up – health professionals’ views on follow-up surgical treatment in gynecological cancer. Acta Oncologica 2015, 54:2, 194-199. | Full text | Included discussion on individualised follow-up, but not PIFU. |
| Girgis et al. ‘This follow-up care would meet my needs, but I would still worry that problems with my health would not be found’: a cross-sectional survey of adult cancer survivors. COSA 39TH annual scientific meeting and IPOS 14TH world congress of psycho-oncology 2012; 5 | Conference abstract | Survey on different FU models, but PIFU not included. |
| Jeppesen MM, Jensen PT, Hansen DG, Christensen RD, Mogensen O. Patient-initiated follow up affects fear of recurrence and healthcare use: a randomised trial in early-stage endometrial cancer. BJOG. 2018;125(13):1705-14. | Full text | Outcomes did not match inclusion criteria (patient preference, satisfaction, acceptability). |
| Kwast et al. Breast cancer follow-up: from the perspective of health professionals and patients. European Journal of Cancer Care 2013; 22, 754–764 | Full text | Focus on purpose and frequency of FU, type of investigations and type of HC professional. No mention of PIFU. |
| Lavery et al. Transforming follow-up: Patients as true partners in a major multi-disciplinary change project. European Journal of Cancer 2013;49:S438 | Conference abstract | Description of working party developing shortened FU pathway for breast cancer patients, including self-referral service. No data on views of patients of professionals on self-referral. |
| Martin et al. Development and Qualitative Evaluation of a Self-Management Workshop for Testicular Cancer Survivor–Initiated Follow-Up. Oncology Nursing Forum 2013; 40(1):E14 | Full text | Discusses experiences of workshop. General aspects of self-management, no focus on PIFU. |
| Megson et al. Patient and clinician attitudes to the implementation of ePRIME (electronic Patient Self-Reported outcomes to Improve cancer Management and patient Experiences)—a remote monitoring pathway for follow-up after ovarian cancer: a qualitative interview study. Psycho-Oncology 2017; 26 (Suppl. 2): 3–9 | Conference abstract | Study aims to explore patient and clinician views on challenges of follow-up on ovarian cancer, in particular views on impact of the ePRIME remote monitoring pathway on self-management and patient empowerment. No information on PIFU specifically. |
| Nugteren et al. Need for general practitioner involvement and eHealth in colon cancer survivorship care: patients’ perspectives. Family Practice 2017; 34 (4): 473–478 | Full text | Focus on GP-led survivorship care and eHealth for self-management of symptoms. No focus on recurrence or PIFU. |
| O’Brien et al. Experiences of follow-up after treatment in patients with prostate cancer:  a qualitative study. BJU International 2010; 106:998–1003 | Full text | Experiences of current follow-up practices -primary, secondary or shared care. |
| Reb et al. Empowering Survivors After Colorectal and Lung Cancer Treatment: Pilot Study of a Self-Management Survivorship Care Planning Intervention. Eur J Oncol Nurs. 2017 August ; 29: 125–134. | Full text | Did not include focus on follow-up for recurrence. |
| Roorda et al. Patients’ preferences for post-treatment breast cancer follow-up in primary care vs. secondary care: a qualitative study. Health Expectations, 18, pp.2192–2201 | Full text | Focus on frequency of FU and GP versus hospital led follow-up. |
| Semple et al. Development and evaluation of a holistic surgical head and neck cancer post-treatment follow-up clinic using touchscreen  technology—Feasibility study. Eur J Cancer Care 2018; 27: e12809 | Full text | Feasibility on using information (from HRQoL tool and holistic item prompt list) to focus routine follow-up consultations. |
| Smith et al. Implementing provider and patient initiated survivorship care planning: Lessons from baseline. Journal of Clinical Oncology 2016; 34(3) Supplement, p 65 | Conference abstract | Abstract of study below. No information related to PIFU. |
| Smith et al. Comparing Web-Based Provider-Initiated and Patient-Initiated  Survivorship Care Planning for Cancer Patients: A Randomized Controlled Trial. JMIR Cancer 2016; 2(2):e12 | Full text | Includes qualitative interviews but relates to initiation and completing of a survivorship care plan by patients or clinicians; discussion of unmet needs. No focus on PIFU. |
| Sterba et al. Development of a Survivorship Needs Assessment Planning Tool for Head and Neck Cancer Survivors and Their Caregivers: A Preliminary Study. J Cancer Surviv 2017; 11(6): 822–832. | Full text | Identification of post-treatment challenges to inform development of a survivorship needs assessment planning tool, and pre-testing of tool. |
| Van Egmond et al. What do patients and dermatologists prefer regarding low-risk basal cell carcinoma follow-up care? A discrete choice experiment. PLOS ONE 2021; | Full text | Treatment as per guideline or with additional FU appts. Not PIFU. |
| Wells M, Semple CJ & Lane C. A national survey of healthcare professionals’ views on models of follow-up, holistic needs assessment and survivorship care for patients with head and neck cancer. European Journal of Cancer Care 2015; 24: 873-83 | Full text | Healthcare professionals’ views and practices in relation to models of  follow-up, HNA and survivorship care for patients with head and neck cancer. No fous on PIFU. |
