# Supplementary material for: Systematic review of patients’ and healthcare professionals’ views on patient‐initiated follow‐up in treated cancer patients
Source: Cancer Med. 2023 Jun 16;12(15):16531–47. doi: 10.1002/cam4.6243 (PMC10469665; doi:10.1002/cam4.6243)
Supplement: Supplementary file 7 — Data S7. [file CAM4-12-16531-s004.docx]

**ENTREQ reporting guidelines**

| **No** | **Item** | **Guide and description** |
| --- | --- | --- |
| **1** | Aim | Last sentence of introduction, p6. |
| **2** | Synthesis methodology | Thematic synthesis as described under analysis on p7. |
| **3** | Approach to searching | Pre-planned as described under ‘searches’ on p6. Sample search strategy provided. |
| **4** | Inclusion criteria | Inclusion/exclusion criteria specified in Table 1. |
| **5** | Data sources | Information sources and time-period described under ‘searches’ on p6. |
| **6** | Electronic Search strategy | Sample search strategy provided. |
| **7** | Study screening methods | Described under ‘Study eligibility criteria and screening’ on p6. |
| **8** | Study characteristics | Presented in Table 2 (and in supplementary material for surveys). |
| **9** | Study selection results | PRISMA flowchart (Figure 1). Reasons for exclusion in supplementary material. |
| **10** | Rationale for appraisal | CASP critical appraisal tool for qualitative studies – to assess conduct, reporting and value of research. |
| **11** | Appraisal items | CASP critical appraisal tool for qualitative studies. For surveys, details on questionnaire design, sampling method, response rate and representativeness of sample were noted. |
| **12** | Appraisal process | Undertaken by one reviewer and checked by a second (p7). Disagreements resolved through discussion. |
| **13** | Appraisal results | Presented in detail in supplementary material, with a summary in the article text (p10 and 14). |
| **14** | Data extraction | Described under ‘data extraction’ and ‘analysis’ (p7). |
| **15** | Software | Covidence and Rayyan used for screening (p6). |
| **16** | Number of reviewers | Described under ‘analysis’ (p7). |
| **17** | Coding | Described under ‘analysis’ (p7). |
| **18** | Study comparison | Data for each study grouped, within themes, under ‘barrier’ or facilitator’. This enabled comparison of barriers and facilitators across studies. Similarities or differences between patients and health care professional views (within themes) were also noted. (See ‘analysis’, p7) |
| **19** | Derivation of themes | Inductive approach to generate new themes. |
| **20** | Quotations | Provided in Table 3. |
| **21** | Synthesis output | Findings presented under five themes derived from the studies (p10 onwards) and put into context of wider literature in the discussion. |
